# Supplementary material for: Ca2+-modulated photoactivatable imaging reveals neuron-astrocyte glutamatergic circuitries within the nucleus accumbens
Source: Nat Commun. 2022 Sep 7;13:5272. doi: 10.1038/s41467-022-33020-6 (PMC9452556; doi:10.1038/s41467-022-33020-6)
Supplement: Supplementary file 1 — Supplementary Information [file 41467_2022_33020_MOESM1_ESM.pdf]

SUPPLEMENTARY INFORMATION

Ca^{2+}-Modulated Photoactivatable Imaging Reveals Neuron-Astrocyte Glutamatergic Circuitries Within The Nucleus Accumbens

Irene Serra[1], Julio Esparza[1], Laura Delgado[1], Cristina Martin-Monteagudo[1], Margalida Puigròs[2], Petar Podlesniy[2]; Ramón Trullás[2], Marta Navarrete[1*]

1. Instituto Cajal, CSIC, Madrid, Spain.
2. Institute for Biomedical Research of Barcelona, CSIC, CIBERNED, Barcelona, Spain.

[*]Correspondence should be addressed to:
Dr. Marta Navarrete

Instituto Cajal

Doctor Arce 37

Madrid 28002, Spain

Phone: +34 915854648
E-mail: mllinas@cajal.csic.es

Includes Supplementary Figures 1-13 and Supplementary Table 1: Statistic and Reproducibility.

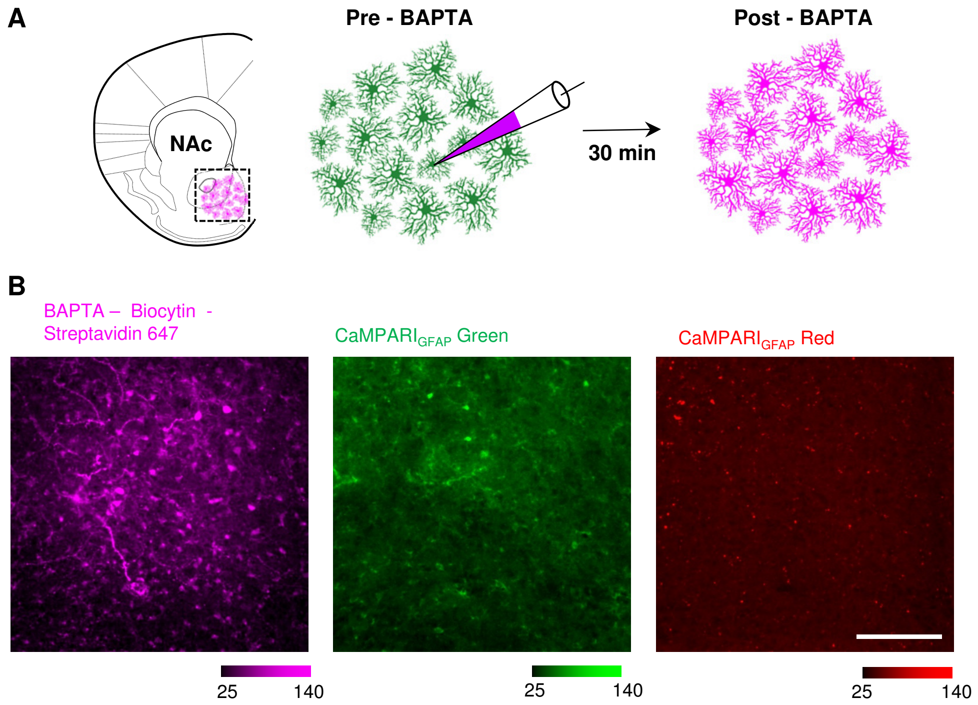

Figure S1. BAPTA-Biocytin loaded astrocytes show no CaMPARI$_{GFAP}$ photoconversion in response to ATP

A Scheme depicting BAPTA-Biocytin dialysis into the astrocytic network.
B Representative confocal images of NAc slices showing BAPTA-Biocytin signal revealed with streptavidine-647 (magenta), CaMPARI$_{GFAP}$ Green and CaMPARI$_{GFAP}$ Red after local application of ATP and 405 nm photoconversion protocol. Scale bar = 100 µm. Source data are provided as a Source Data file.

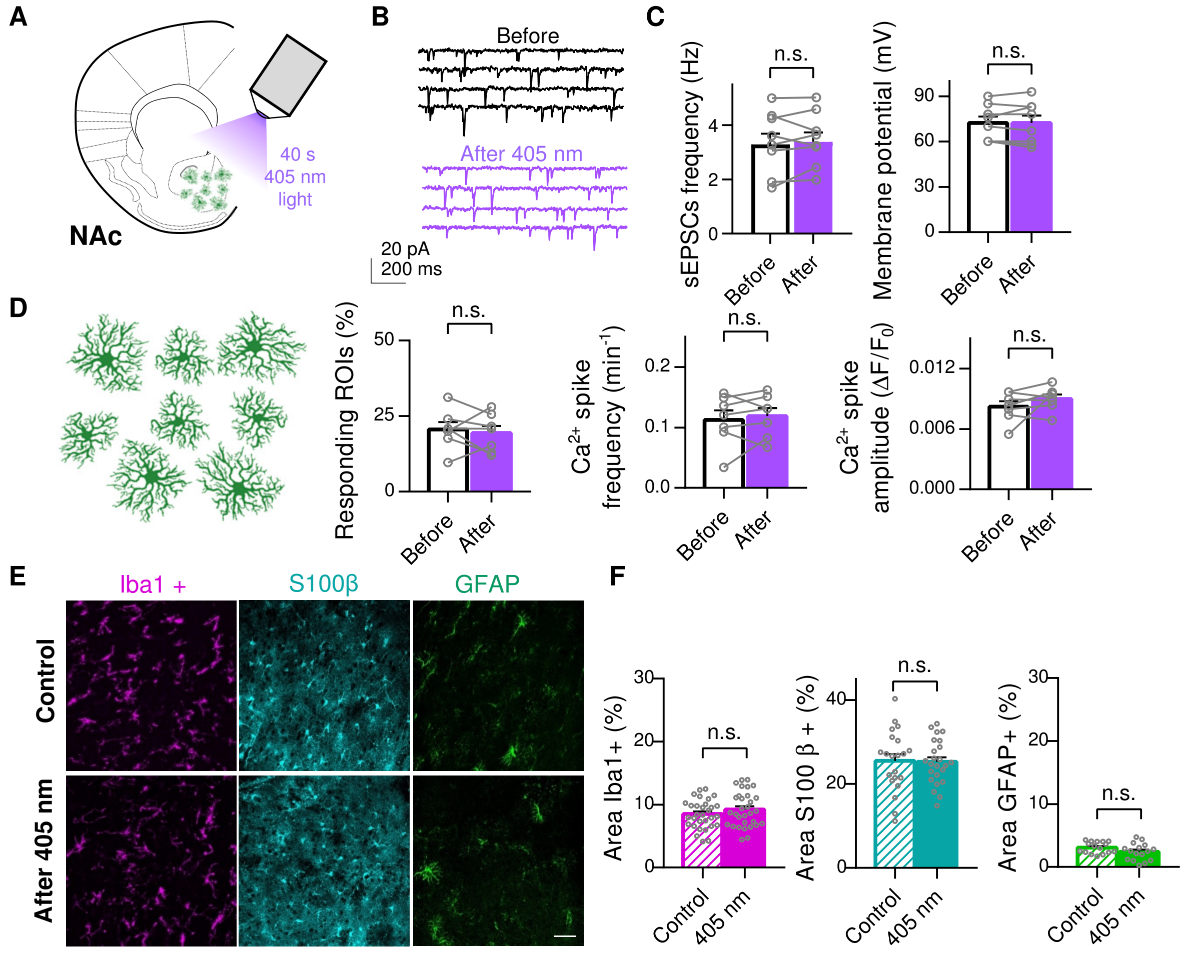

Figure S2. Violet light (405 nm) photoconversion protocol does not induce cell excitability or tissue damage.

A Scheme of the photoconversion protocol in which 40s of violet light (405 nm) is full field applied to NAc slices. **B** Representative traces of NAc neuron´s spontaneous excitatory postsynaptic currents (sEPSCs) registered before and after 405 nm photoconversion protocol. **C** Quantification of sEPSCs frequency (left; 3.29 ± 0.4 Hz before vs 3.38 ± 0.4 Hz after; $^{n.s.}p = 0.53$) and membrane potential (right; 72.4 ± 4.18 |mV| before vs 72.3 ± 4.89 |mV| after; $^{n.s.}p = 0.92$) shows no changes in NAc neuron´s excitability (8 cells, 2 mice). Two-tailed paired t-test. **D** CaMPARI$_{GFAP}$ green infected astrocytes (left) in which calcium response (middle left; 20.6 ± 2.46 % before vs 19.4 ± 2.37 % after; $^{n.s.}p = 0.64$), Ca^{2+} spike frequency (middle right; 0.113 ± 0.02 min-1 before vs 0.119 ± 0.01 min-1 after; $^{n.s.}p = 0.54$) and Ca^{2+} spike amplitude (right; 0.008 ± 6.10-4 ΔF/F0 before vs 0.009 ± 5.10-4 ΔF/F0 after; $^{n.s.}p = 0.23$) is assessed before and after 405 nm light photoconversion protocol showing no changes in NAc astrocytes excitability (450 ROIs; 7 slices, 1 mouse). Two-tailed paired t-test. **E** Representative confocal images showing immunolabeling of microglia (Iba1 marker; magenta) and astrocytes (S100β marker; cyan and GFAP marker; green) in control NAc slices and in slices illuminated with 405 nm photoconversion protocol. Scale bar = 50 µm. **F** Labeled area (%) quantification for Iba1 (magenta bars; 8.5 ± 0.41 % before vs 9.23 ± 0.5 % after; n.s.p = 0.26; 31 fields, 3mice), S100β (cyan bars; 25.5 ± 1.59 % before vs 25.3 ± 1.08 % after; n.s.p = 0.88; 24 fields, 3 mice) and GFAP markers (green bars; 3.1 ± 0.23 % before vs 2.42 ± 0.34 % after; n.s.p = 0.1; 16 fields, 2 mice) showing no differences among conditions. Two-tailed unpaired t-test. Error bars express SEM. Source data are provided as a Source Data file.

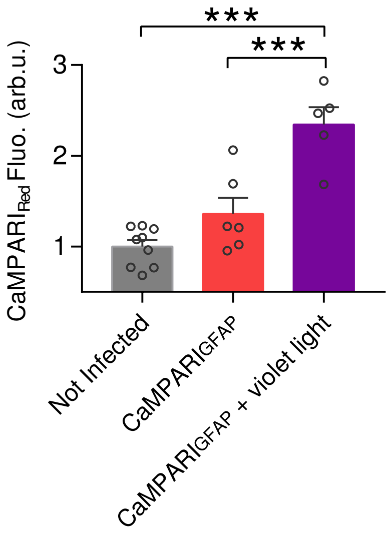

Figure S3. Spontaneous $CaMPARI_{GFAP}$ photoconversion.
$CaMPARI_{Red}$ fluorescence (arb.u.) quantification in not-infected nucleus accumbens tissue (gray bar; 1 a.u.; 9 slices, 3 mice), in tissue infected with $CaMPARI_{GFAP}$ but without photoconversion (red bar; 1.36 ± 0.18 a.u.; 6 slices, 3 mice), and in tissue infected with $CaMPARI_{GFAP}$ after 40 s of 405 nm light (purple bar; 2.35 ± 0.19 a.u.; 5 slices, 3 mice). Values normalized to the average red signal obtained from not infected tissue. One-way ANOVA, Holm-Sidak test for multiple comparisons, ***: $p < 0.001$. Error bars express SEM. Source data are provided as a Source Data file.

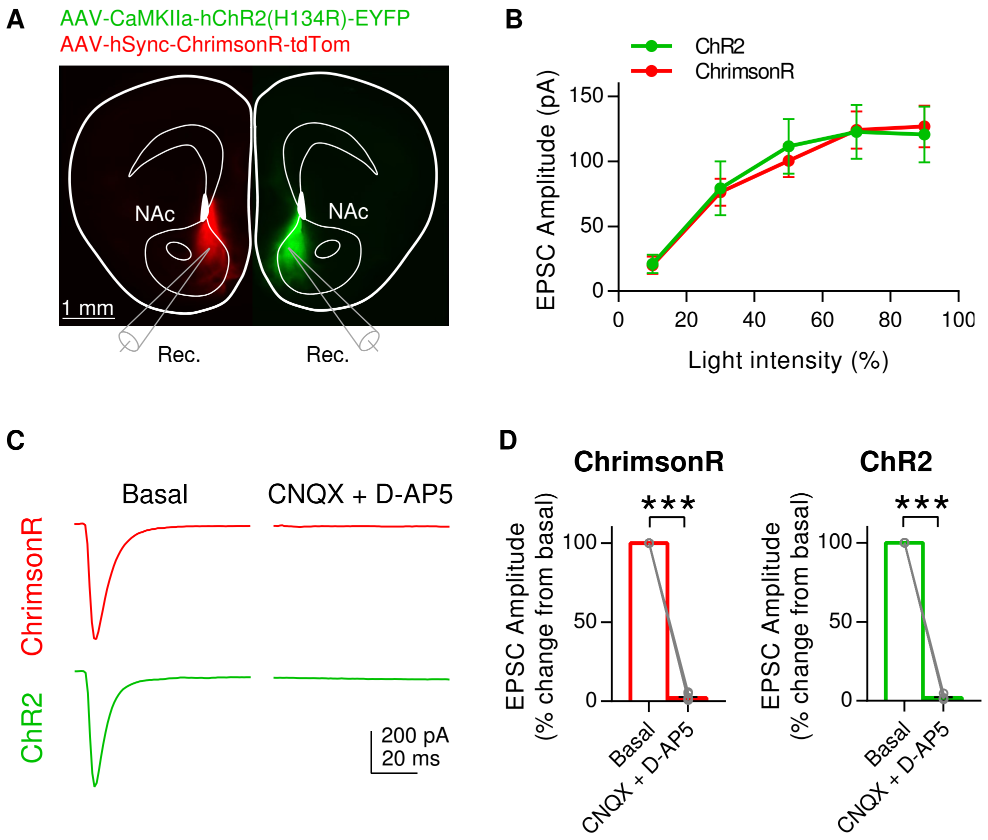

Figure S4. Channelrhodopsin (ChR2) or ChrimsonR activate equally glutamatergic afferents in the nucleus accumbens (NAc).

A Representative NAc slices with opsin-infected afferents expressing ChrimsonR (red) and ChR2 (green). Scale bar =1 mm. **B** Dose-response curve showing the relationship between EPSC amplitudes (pA) triggered in NAc neurons, and the light intensity (%) used to activate ChR2 (green line; 9 cells, 5 mice) and ChrimsonR (red line; 9 cells, 4 mice). Both opsins elicited similar EPSC amplitude at different light intensities, showing maximum responses above 70%. Two-way ANOVA, $p = 0.88$. **C** Representative traces showing optostimulated EPSC amplitudes in basal condition and after extracellular perfusion of glutamate receptor antagonists (CNQX and D-AP5). **D** EPSC amplitude recordings from NAc neurons in response to optostimulation of afferents expressing ChrimsonR (red bars; 2.12 ± 0.55 % change from basal; 10 cells, 1 mouse) and ChR2 (green bars; 1.83 ± 0.44 % change from basal; 8 cells, 1 mouse) in basal condition and after extracellular perfusion of a glutamate receptor antagonist. EPSC amplitude values are normalized to basal response in each neuron. Two-tailed paired t-test, ***: $p < 0.001$. Error bars express SEM. Source data are provided as a Source Data file.

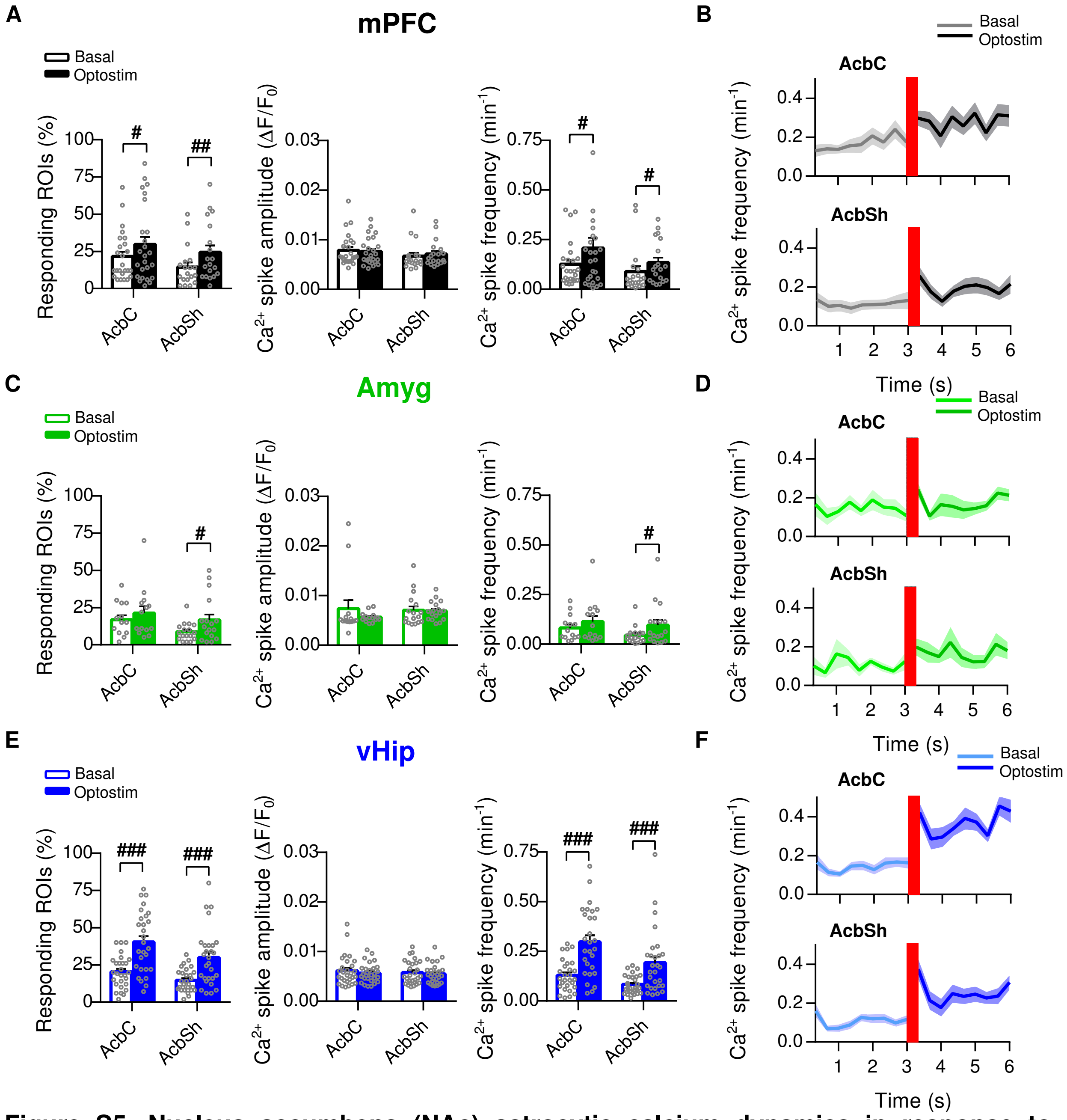

Figure S5. Nucleus accumbens (NAc) astrocytic calcium dynamics in response to optostimulation.

A, C, E Study of astrocytic basal activities and after afferent optostimulation, showing average response, change of Ca^{2+} spike frequencies and amplitudes in the core (AcbC) and shell (AcbSh) of the NAc in medial prefrontal cortex (mPFC black; AcbC 25 slices, AcbSh 19 slices, 8 mice), basolateral amygdala (Amyg green; AcbC 14 slices, AcbSh 17 slices, 7 mice), and ventral hippocampus (vHip blue; AcbC 28 slices, AcbSh 28 slices, 8 mice), respectively. Two-tailed paired t-test, #: $p < 0.05$; ##: $p < 0.01$; ###: $p < 0.001$. Between subregions, two-way ANOVA. For more detail see Statistics and Reproducibility file. Note that for the three glutamatergic inputs astrocytic responses are due to changes in the frequency of Ca^{2+} spikes without significant changes in the amplitude of those responses. All error bars express SEM.
B, D, E Temporal study of astrocytic Ca^{2+} spike frequency in AcbC and AcbSh subregions, 3 min bin before and after optostimulation of mPFC (black), Amyg (green) and vHip (blue) afferents, respectively. Red bar indicates optostimulation period. Error bars express SEM. Source data are provided as a Source Data file.

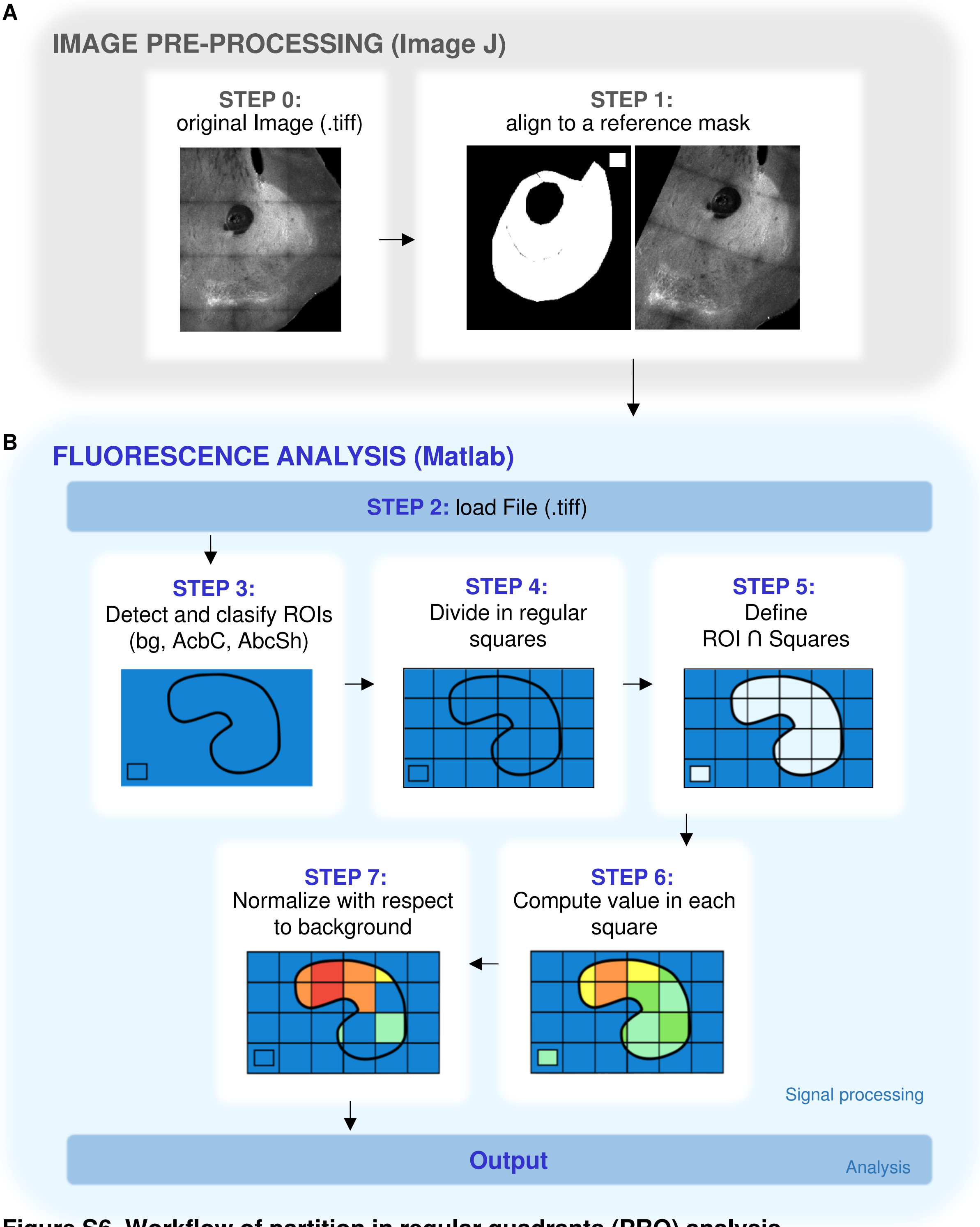

Figure S6. Workflow of partition in regular quadrants (PRQ) analysis.
A Data pre-processing. Raw z-stacks (10 steps – 10 µm) are aligned to a reference mask to maintain the anatomical structure across different samples. **B** Signal processing and analysis of fluorescence. Z-stack average image is calculated and divided in a regular grid (50 µm x 50 µm pixels) in which the AcbC, AcbSh and background areas are defined (steps 3 to 5). Fluorescence values are assigned to each pixel according to the mean fluorescence signal inside it, and the entire grid is normalized to a background (bg) signal computed from a region outside the NAc (steps 6 and 7). Output grid is used for analysis. Source data are provided as a Source Data file.

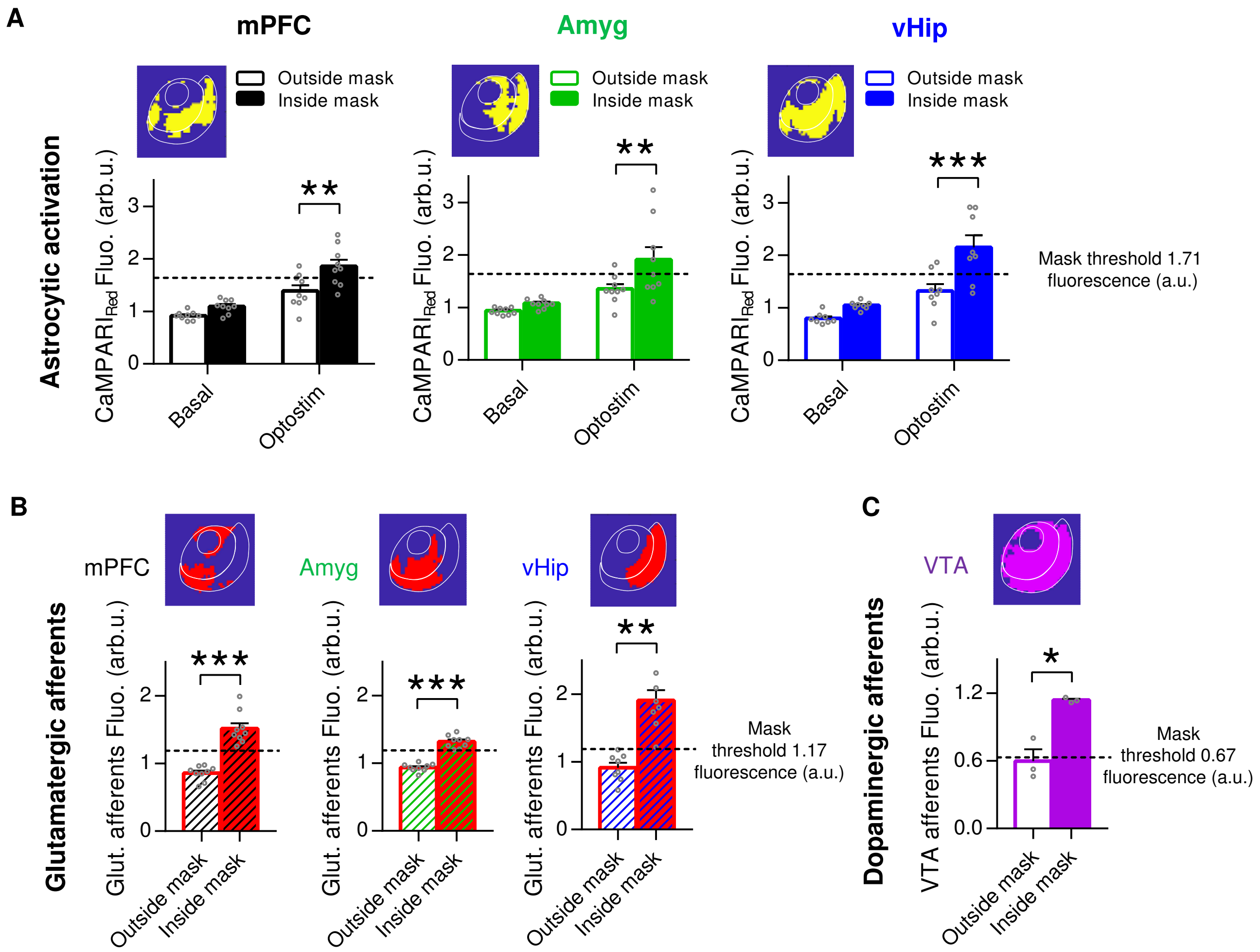

Figure S7. Inside and outside fluorescence of PRQ binary masks verifies the activation areas defined by k-mean clustering

A CaMPARI$_{Red}$ fluorescence signal (arb.u.) quantified inside (solid bars) and outside (white bars) the activation masks in basal condition and after afferent optostimulation of mPFC, Amyg and vHip respectively. Insets showing in yellow the active NAc astrocytes binary masks defined by k-mean clustering in response to mPFC, Amyg and vHip, respectively. Discontinuous line indicates the activation threshold, defined at 1.71 fluorescence (arb.u.). Note that statistical differences between inside-outside regions are only found after optostimulation, indicating increased astrocytic activity gathered in the area defined by the mask. Two-way ANOVA, Holm-Sidak test for multiple comparisons, **: $p < 0.01$; ***: $p < 0.001$. **B** Glutamatergic afferents fluorescence signal (arb.u.) quantified inside (solid bars) and outside (slashed bars) of the binary masks defined by k-mean clustering for mPFC, Amyg and vHip, respectively. Insets showing in red the binary mask for mPFC, Amyg and vHip innervation patterns, respectively. Discontinuous line indicates the activation threshold, defined at 1.17 fluorescence (arb.u.). Two-tailed paired t-test, **: $p < 0.01$; ***: $p < 0.001$. **C** VTA afferents fluorescence signal (arb.u.) quantified inside (solid bars) and outside (slashed bars) of the binary masks defined by k-mean clustering. Insets showing in red the binary mask for mPFC, Amyg and vHip innervation patterns, respectively. Discontinuous line indicates the activation threshold, defined at 0.67 fluorescence (arb.u.). Two-tailed paired t-test, *: $p < 0.05$. For more detail see Statistics and Reproducibility file. Error bars express SEM. Source data are provided as a Source Data file.

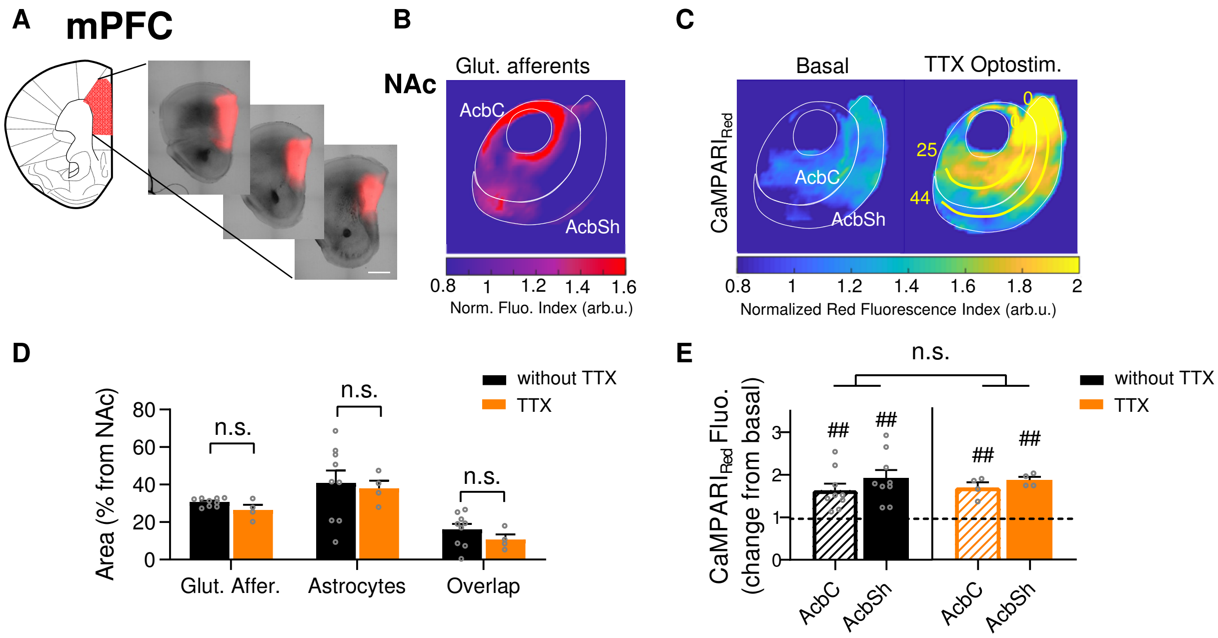

Figure S8. NAc astrocytic response to medial prefrontal cortex (mPFC) afferents in presence of TTX.

A Scheme and representative slices showing ChR2-EYFP-expression after virus injection into the mPFC. Scale bar = 1 mm. **B** Average PRQ image showing mPFC glutamatergic innervation pattern. **C** Average PRQ image showing astrocytic activity pattern in the NAc in presence of TTX (1 µM), in basal and optostimulated conditions. **D** Area (% from NAc) quantification of the glutamatergic afferents (26.6 ± 2.6 % with TTX *vs* 30.8 ± 0.75 % without TTX; p = 0.90), the associated astrocytic response (38 ± 4.12 % with TTX *vs* 40.9 ± 6.66 % without TTX; p = 0.97) and the spatial overlap between the two (10.7 ± 2.72 % with TTX *vs* 16.2 ± 2.94 % without TTX; p = 0.81) in presence of TTX (orange bars; 4 slices, 2 mice) and without TTX (black bars; 9 slices, 6 mice). Two-way ANOVA, Holm-Sidak test for multiple comparisons, n.s: $p > 0.05$. **E** Quantification of astrocytic $\text{CaMPARI}_{\text{Red}}$ fluorescence (arb.u.) in response to optostimulation in presence of TTX (orange bars; 1.67 ± 0.12 arb.u. ni AcbC and 1.84 ± 0.07 arb.u. ni AcbSh; 4 pairs basal-stim slices, 2 mice) and without TTX (black bars; 1.64 ± 0.16 arb.u. ni AcbC and 1.92 ± 0.19 arb.u. ni AcbSh; 9 pairs basal-stim slices, 6 mice) (between groups; p = 0.45). Two-way ANOVA, n.s: $p < 0.05$. Note that there are no differences between conditions regarding both, activation mask's area and intensity of $\text{CaMPARI}_{\text{Red}}$ signal. Error bars express SEM. Source data are provided as a Source Data file.

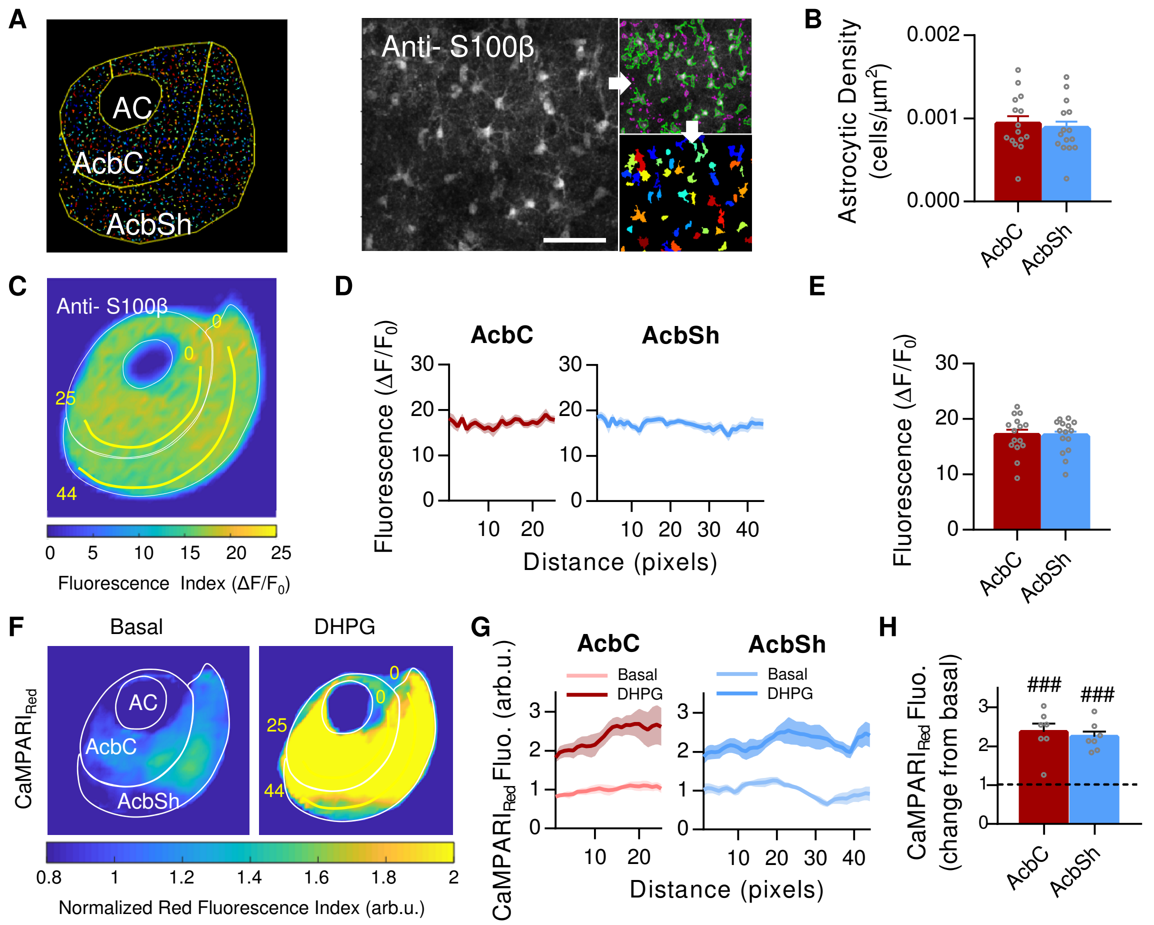

Figure S9. Homogeneous cell distribution and activity of mGluR5/1 in nucleus accumbens (NAc) astrocytes.

A Left, representative NAc image showing the result of individual cells automatic segmentation used for quantification. Right, detail of the segmentation process based on the identification of astrocytes labeled with the specific marker S100β. Scale bar = 50 µm. **B** Quantification of astrocytic density at the AcbC (red bar; $9.4 \times 10^{-4} \pm 8.8 \times 10^{-5}$ cells/µm^2) and AcbSh (blue bar; 8.8 x10-4 ± 8.2 x10-5 cells/µm^2) subregions ($p = 0.61$; 15 slices, 3 mice). Two-tailed unpaired t-test. **C** Average PRQ image showing astrocytic distribution pattern in the NAc determined by S100β fluorescence labeling ($\Delta F/F_0$). Yellow lines starting from pixel 0 in each subregion were used for quantification (pixel = 50 µm^2). **D** S100β fluorescence ($\Delta F/F_0$) *vs* distance (pixels) quantifying astrocytic distribution across yellow lines. **E** Average spatial fluorescence ($\Delta F/F_0$) at AcbC (red bar; 17.2 ± 0.9 $\Delta F/F_0$) and AcbSh (blue bar; 16.9 ± 0.78 $\Delta F/F_0$) ($p = 0.84$; 15 slices, 3 mice). Two-tailed unpaired t-test. Note that astrocytic marker fluorescence signal is constant across the nucleus, indicating a homogenous distribution of cells. **F** Average PRQ image showing astrocytic activation pattern in the NAc in basal and after bath perfusion of the mGluR5/1 receptors agonist (DHPG, 50 µM). Yellow lines starting from pixel 0 in each subregion were used for quantification (pixel = 50 µm^2). **G** $CaMPARI_{Red}$ fluorescence (a.u.) *vs* distance (pixels) quantifying astrocytic activation across space. **H** Average $CaMPARI_{Red}$ spatial fluorescence (arb.u.) in DHPG-stimulated condition *vs* basal at the AcbC (red bar; 2.37 ± 0.21; $p = 0.0007$) and AcbSh (blue bar; 2.24 ± 0.14; $p = 0.0001$) (between groups $p = 0.64$; 7 pairs basal-stim slices, 4 mice). One-sample t-test, ###: $p < 0.001$; two-tailed unpaired t-test; $p > 0.05$. Note that astrocytic activation is constant across the nucleus, indicating a homogenous activation of mGluR5/1 receptors. Error bars express SEM. Source data are provided as a Source Data file.

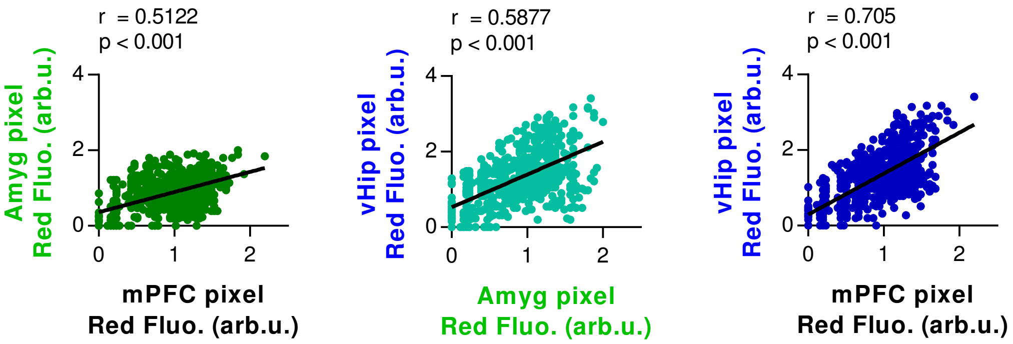

Figure S10. Positive correlation between NAc astrocytic-networks indicates no spatial segregation of the astrocytic response between pathways.

Spatial comparison NAc astrocytic responses triggered by different glutamatergic pairs (see also Fig. 5A). Pixel values show the astrocytic activation given by $CaMPARI_{Red}$ fluorescence (arb.u.) for an experimental condition (Amyg, vHip and mPFC) in a specific pixel area (50 μm^2). Note that in all cases there is a positive correlation, indicating that pixels occupying the same space show activation in response to the three pathways. Pearson r correlation (two-tailed). Source data are provided as a Source Data file.

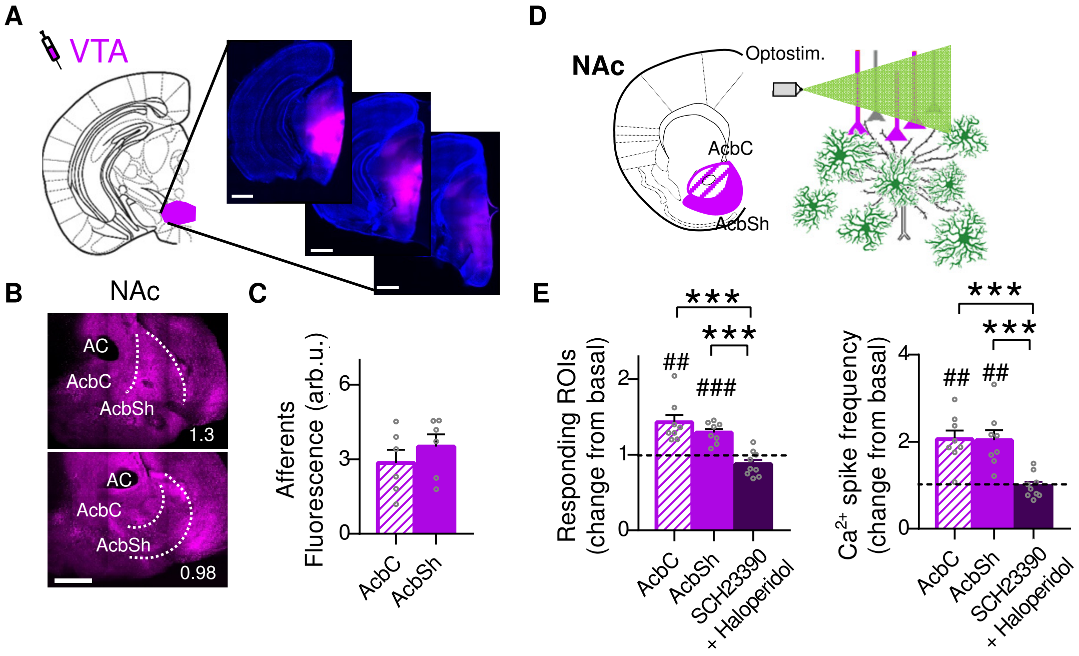

Figure S11. Pathway-specific NAc astrocyte activity in response to ventral tegmental area (VTA) inputs.

A Scheme and representative brain slices showing opsin expression in VTA. Scale bar = 1 mm. **B** Coronal brain slices showing opsin expression of VTA axons coming to the NAc. Scale bar = 500 µm. **C** Quantification of VTA afferents' fluorescence (arb.u.) at AcbC (2.86 ± 0.53 arb.u.) and AcbSh (3.51 ± 0.49 arb.u.), showing the specific innervation profile of VTA (6 infections, 3 mice). Two-tailed unpaired t-test; $p = 0.38$. **D** Scheme of astrocytic Ca^{2+} dynamics, monitored by real-time imaging of $CaMPARI_{GFAP}$ Green fluorescence, in response to VTA axons. **E** Left, proportion of ROIs responding to VTA-afferent optostimulation at the AcbC (slashed bar; 1.43 ± 0.1 change from basal) and AcbSh (solid bar; 1.29 ± 0.05 change from basal) in control condition (800 ROIs; 8 slices, 5 mice) and in presence of dopamine antagonist haloperidol (10 µM) and SCH 23390 (10 µM) (dark purple bar; 0.88 ± 0.75 change from basal; AcbC and AcbSh pooled together, 9 slices, 2 mice). Right, average change of astrocytic Ca^{2+} spike frequency in response to optostimulation at the AcbC (slashed bar; 2.06 ± 0.2 change from basal) and AcbSh (solid bar; 2.04 ± 0.23 change from basal) in control condition (8 slices, 5 mice) and in presence of haloperidol (10 µM) and SCH (10 µM) 23390 (dark purple bar; 0.99 ± 0.1 change from basal; AcbC and AcbSh pooled together, 9 slices, 2 mice). One-sample t-test, ##: $p < 0.01$; ###: $p < 0.001$; one-way ANOVA, Holm-Sidak test for multiple comparisons, ***: $p < 0.001$. Error bars express SEM. Source data are provided as a Source Data file.

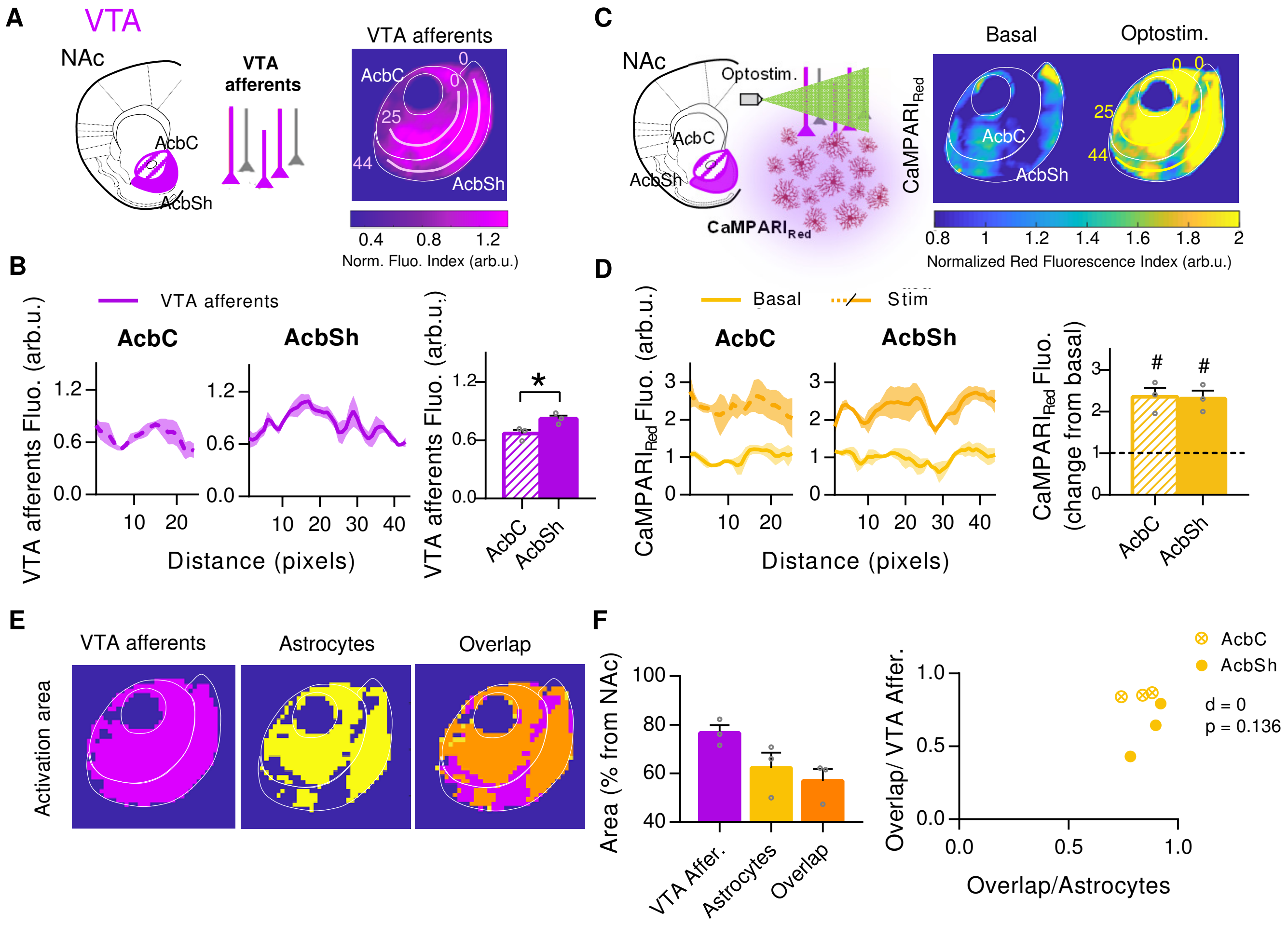

Figure S12. Pathway-specific NAc astrocyte activity in response to ventral tegmental area (VTA) inputs.

A Left, scheme of opsin-transfected VTA afferents in the NAc. Right, average PRQ image showing VTA innervation pattern. White lines starting from pixel 0 in each subregion were used for quantification (pixel = 50 μm^2). **B** Left, VTA afferents fluorescence (arb.u.) *vs* distance (pixels) quantifying VTA innervation across white lines. Right, average VTA afferents spatial fluorescence (arb.u.) in the AcbC (slashed bar; 0.67 ± 0.04 arb.u.) and AcbSh (solid bar; 0.82 ± 0.03 arb.u.) (3 slices, 3mice). Two-tailed unpaired t-test, *: p = 0.04. **C** Left, scheme of astrocytic Ca^{2+} activity, measured by CaMPARI$_{GFAP}$ Red, in response to VTA axons. Right, average PRQ image showing astrocytic activation pattern in the NAc in basal and optostimulated conditions. Yellow lines starting from pixel 0 in each subregion were used for quantification (pixel = 50 μm^2). **D** Left, CaMPARI$_{Red}$ fluorescence (arb.u.) *vs* distance (pixels) quantifying astrocytic activation across yellow lines. Right, average CaMPARI$_{Red}$ spatial fluorescence (arb.u.) in optostimulated condition with respect to basal, from control experiments in the AcbC (slashed bar; 2.35 ± 0.22 arb.u.) and AcbSh (solid bar; 2.31 ± 0.17 arb.u.) (3 pairs basal-stim slices, 3 mice). One-sample t-test, #: $p < 0.05$; two-tailed unpaired t-test, p = 0.90. **E** Masks of VTA afferents (purple) and astrocyte activation area (yellow) defined by a k-mean clustering. In orange, the overlap area between the two. **F** Left, Area (% from NAc) quantification of the spatial overlap (orange bar; 57.01 ± 4.85 %) between VTA afferents (purple bar; 76.7 ± 3.12 %) and active astrocytes (yellow bar; 62.2 ± 6.30 %) (3 slices, 3 mice). Note that there is a high degree of colocalization between astrocytic responses and areas with strong VTA dopaminergic innervation. One-way ANOVA, Holm-Sidak test for multiple comparisons, $p > 0.05$. Error bars express SEM. Source data are provided as a Source Data file.

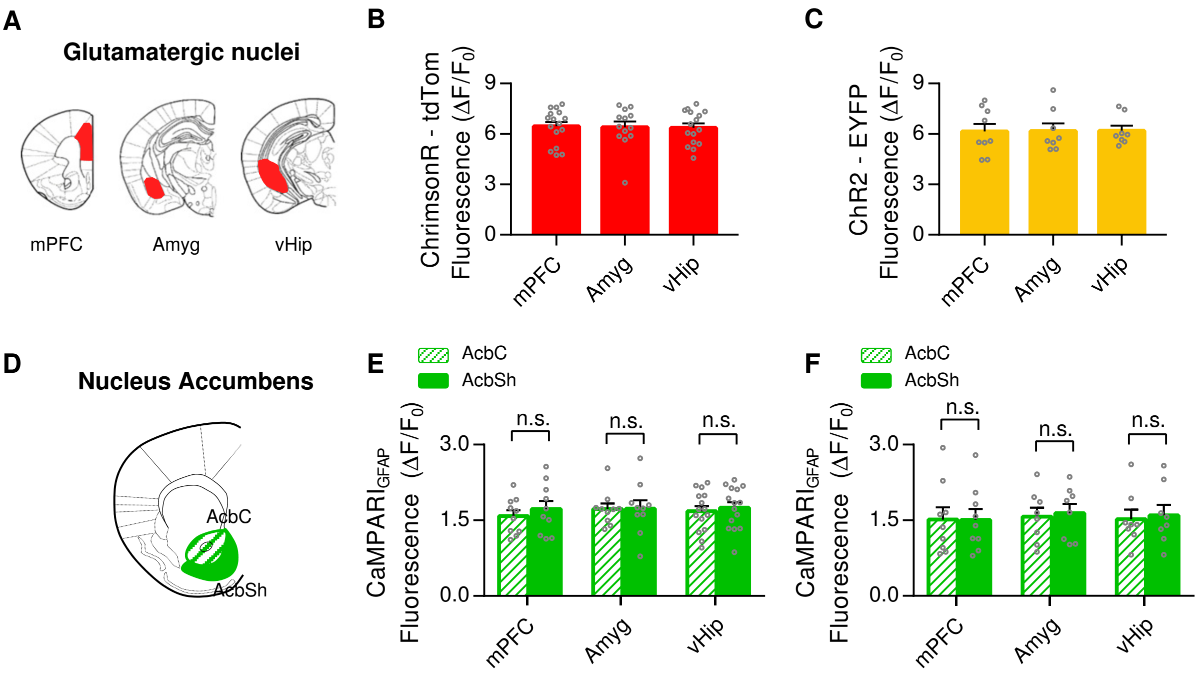

Figure S13. AAV-transfection degree for ChR2/ChrimsonR opsins and CaMPARI$_{GFAP}$. **A** Scheme of opsin-injection site for the three glutamatergic nuclei. **B** ChrimsonR-tdTom fluorescence ($\Delta F/F_0$) quantificated at the virus injection site for mPFC (6.45 ± 0.26 $\Delta F/F_0$; 16 infections, 8 mice), Amyg (6.40 ± 0.34 $\Delta F/F_0$; 13 infections, 7 mice) and vHip (6.37 ± 0.25 $\Delta F/F_0$; 16 infections, 8 mice; $p = 0.98$). One-way ANOVA. **C** ChR2-EYFP fluorescence ($\Delta F/F_0$) measured at the injection site for mPFC (6.15 ± 0.45 $\Delta F/F_0$; 9 infections, 6 mice), Amyg (6.18 ± 0.44 $\Delta F/F_0$; 8 infections, 6 mice) and vHip (6.18 ± 0.31 $\Delta F/F_0$; 8 infections, 6 mice; $p > 0.99$). One-way ANOVA. **D** Scheme of CaMPARI$_{GFAP}$ injection at the nucleus accumbens (NAc). **E** Representative quantification of CaMPARI$_{GFAP}$ fluorescence ($\Delta F/F_0$), mPFC (AcbC, 1.58 ± 0.26 $\Delta F/F_0$ and AcbSh, 1.72 ± 0.16 $\Delta F/F_0$; between subregions, $p = 0.85$; 10 infections, 5 mice), Amyg (AcbC, 1.73 ± 0.11 $\Delta F/F_0$ and AcbSh, 1.73 ± 0.16 $\Delta F/F_0$; between subregions, $p > 0.99$; 10 infections, 5 mice) and vHip (AcbC, 1.68 ± 0.1 $\Delta F/F_0$ and AcbSh, 1.75 ± 0.11 $\Delta F/F_0$; between subregions, $p = 0.95$; 15 infections, 8 mice). Two-way ANOVA, Holm-Sidak test for multiple comparisons; n.s; $p > 0.05$. **F** Quantification of CaMPARI$_{GFAP}$ fluorescence ($\Delta F/F_0$) indicating equivalent AAV- transfection degree of those slices used for photoconversion experiments ($p = 0.9$); mPFC (AcbC, 1.52 ± 0.24 $\Delta F/F_0$ and AcbSh, 1.51 ± 0.21 $\Delta F/F_0$; between subregions, $p > 0.99$; 9 infections, 6 mice), Amyg (AcbC, 1.57 ± 0.18 $\Delta F/F_0$ and AcbSh, 1.65 ± 0.19 $\Delta F/F_0$; between subregions, $p > 0.99$; 8 infections, 6 mice) and vHip (AcbC, 1.52 ± 0.19 $\Delta F/F_0$ and AcbSh, 1.6 ± 0.21 $\Delta F/F_0$; between subregions, $p > 0.99$; 8 infections, 6 mice). Two-way ANOVA, Holm-Sidak test for multiple comparisons; n.s; $p > 0.05$. Error bars express SEM. Source data are provided as a Source Data file.

Supplementary Table 1. Statistics and Reproducibility			
**Figure**	**Samples Number (n)**	**Statistical Analysis**	
**1B** (CaMPARI colocalization)	n = 8 fields, N = 2 mice	**Two-way ANOVA :**<br>CaMPARI+; F (1, 12) = 7586; p < 0.001<br><br>Holm-Sidak's multiple comparisons test<br>S100+ vs Neun+<br>$CaMPARI_{GFAP}$ Green; p < 0.001 (t = 74.37)<br>$CaMPARI_{GFAP}$ Red; p < 0.001 (t = 52.56)	
**1F** (CaMPARI Real-time Ca2+)	Control; n = 9 slices, N = 2 mice<br>Thapsigargin; n = 6 slices, N = 2 mice<br>BAPTA; n = 2 slices, N = 1 mouse	**One-way ANOVA :**<br>Responding ROIs; F (2, 14) = 1641; p < 0.001<br><br>Holm-Sidak's multiple comparisons test<br>Control vs Thapsigargin; p < 0.001 (t = 52.6)<br>Control vs BAPTA; p < 0.001 (t = 36.1)<br>Thapsigargin vs BAPTA; p = 0.57 (t = 0.582)	
**2B** (Afferents fluorescence)	n = 11 infections, N = 6 mice	**Unpaired t-test**<br>Two-tailed p value<br>AcbC vs AcbSh; p = 0.02 (t = 2.528)	
**2D** (Affer. Fluo vs EPSCs amplitude)	n = 20 cells, N = 3	**Pearson r correlation**<br>Projection Fluo. Vs EPSCs Amplitude<br>r = 0.7278<br>R squared = 0.5297<br>Two-tailed p value; p < 0.001	
**2E** (EPSCs amplitude)	AcbC; n = 8 cells, N = 4 mice<br>AcbSh; n = 15 cells, N = 4 mice	**Unpaired t-test Welch-corrected**<br>Two-tailed p value<br>AcbC vs AcbSh; p = 0.002 (t = 4.454)	
**2H** (Calcium dynamics astrocytes)	AcbC; n = 25 slices, N = 8 mice<br>AcbSh; n = 19 slices, N = 8 mice<br>MPEP; n = 4 AcbC+AcbSh, 4 slices, N = 2 mice	**One-way ANOVA; Responding ROIs**<br>Responding ROIs; F (2, 45) = 3.692, p = 0.03<br><br>Holm-Sidak's multiple comparisons test<br>AcbC vs AcbSh; p = 0.08 (t = 2.302)<br>AcbC vs MPEP; p = 0.41 (t = 0.8383)<br>AcbSh vs MPEP; p = 0.08 (t = 2.094)<br><br>**One-way ANOVA; Ca^{2+} frequency**<br>Ca^{2+} frequency; F (2, 45) = 2.896, p = 0.07<br><br>Holm-Sidak's multiple comparisons test<br>AcbC vs AcbSh; p = 0.16 (t = 1.955)<br>AcbC vs MPEP; p = 0.38 (t =0.8833)<br>AcbSh vs MPEP; p = 0.16 (t = 1.946)	**One sample t-test; Responding ROIs**<br>Change from basal (= 1)<br><br><br>AcbC; p = 0.0228 (t = 2.434)<br>AcbSh; p = 0.0033 (t = 3.386)<br>MPEP; p = 0.4178 (t = 0.9371)<br><br>**One sample t-test; Ca^{2+} frequency**<br>Change from basal (= 1)<br><br><br>AcbC; p = 0.0128 (t = 2.69)<br>AcbSh; p = 0.0062 (t = 3.096)<br>MPEP; p = 0.1939 (t = 1.668)
**3B** (PRQ Glutamatergic afferents)	Optostim; n = 9 slices, N = 6 mice	**Unpaired t-test Welch-corrected - optostim slices**<br>Two-tailed p value<br><br>AcbC vs AcbSh; p < 0.001 (t = 13.59)	**One sample t-test - optostim slices**<br>Change from 1<br><br>AcbC; p < 0.001 (t = 32.07)<br>AcbSh; p = 0.7779 (t = 0.2917)
**3D** (PRQ Astrocytes)	Basal; n = 9 slices, N = 6 mice<br>Optostim; n = 9 slices, N = 6 mice<br><br>MPEP basal; n = 8 AcbC+AcbSh, 4 slices, N = 2 mice<br>MPEP optostim; n = 8 AcbC+AcbSh, 4 slices, N = 2 mic	**One-way ANOVA - optostim slices**<br>F (2, 23) = 8.282, p = 0.002<br><br>Holm-Sidak's multiple comparisons test<br>AcbC vs AcbSh; p = 0.24 (t = 1.216)<br>AcbC vs MPEP; p = 0.02 (t = 2.806)<br>AcbSh vs MPEP; p = 0.002 (t = 3.986)	**One sample t-test - optostim slices**<br>Change from basal (= 1)<br><br><br>AcbC; p = 0.0035 (t = 4.079)<br>AcbSh; p = 0.0014 (t = 4.768)<br>MPEP; p = 0.8354 (t = 0.2156)
**3F** left (Activation masks)	Optostim; n = 9 slices, N = 6 mice	**One-way ANOVA - optostim slices**<br>%Area; F (2, 24) = 8.628, p = 0.002<br><br>Holm-Sidak's multiple comparisons test<br>Glut. Affer. vs Overlap; p = 0.04 (t = 2.441)<br>Glut. Affer. vs Astrocytes; p = 0.1 (t = 1.691)<br>Overlap vs Astrocytes; p = 0.001 (t = 4.131)	
**3F** right (AcbC-AcbSh bivariate)	Optostim; n = 9 slices, N = 6 mice	**MANOVA - optostim slices**<br>AcbC vs Acbsh<br>d = 0, p = 0.586	
**4B** (Afferents fluorescence)	n = 13 infections, N = 7 mice	**Unpaired t-test Welch-corrected**<br>Two-tailed p value<br>AcbC vs AcbSh; p = 0.63 (t = 0.4879)	
**4D** (Affer. Fluo vs EPSCs amplitude)	n = 15 cells, N = 2 mice	**Pearson r correlation**<br>Projection Fluo. Vs EPSCs Amplitude<br>r = 0.5717<br>R squared = 0.3268<br>Two-tailed p value; p = 0.03	
**4E** (EPSCs amplitude)	AcbC; n = 11 cells, N = 3 mice<br>AcbSh; n = 25 cells, N = 4 mice	**Unpaired t-test**<br>Two-tailed p value<br>AcbC vs AcbSh; p = 0.54 (t = 0.6149)	

**4H** (Calcium dynamics astrocytes)	MPEP; n = 8 AcbC+AcbSh, 5 slices, N = 2 mice	Holm-Sidak's multiple comparisons test<br>AcbC vs AcbSh; p = 0.36 (t = 1.367)<br>AcbC vs MPEP; p = 0.73 (t = 0.3475)<br>AcbSh vs MPEP; p = 0.36 (t = 1.51)<br><br>**One-way ANOVA; Ca^{2+} frequency**<br>Ca^{2+} frequency; F (2, 36) = 3.647, p = 0.04<br><br>Holm-Sidak's multiple comparisons test<br>AcbC vs AcbSh; p = 0.08 (t = 2.162)<br>AcbC vs MPEP; p = 0.63 (t = 0.4821)<br>AcbSh vs MPEP; p = 0.08 (t = 2.319)	 AcbC; p = 0.0498 (t = 2.163)<br>AcbSh; p = 0.015 (t = 2.723)<br>MPEP; p = 0.3694 (t = 0.9592)<br><br>**One sample t-test; Ca^{2+} frequency**<br>Change from basal (= 1)<br><br><br>AcbC; p = 0.0401 (t = 2.281)<br>AcbSh; p = 0.0093 (t = 2.956)<br>MPEP; p = 0.1027 (t = 1.876)
**5B** (PRQ Glutamatergic afferents)	Optostim; n = 9 slices, N = 6 mice	**Unpaired t-test - optostim slices**<br>Two-tailed p value<br><br>AcbC vs AcbSh; p = 0.04 (t = 2.246)	**One sample t-test - optostim slices**<br>Change from 1<br><br>AcbC; p = 0.0021 (t = 4.479)<br>AcbSh; p = 0.017 (t = 3.002)
**5D** (PRQ Astrocytes)	Basal; n = 9 slices, N = 6 mice<br>Optostim; n = 9 slices, N = 6 mice<br><br>MPEP basal; n = 8 AcbC+AcbSh, 4 slices, N = 2 mice<br>MPEP optostim; n = 8 AcbC+AcbSh, 4 slices, N = 2 mic	**One-way ANOVA - optostim slices**<br>F (2, 23) = 6.033, p = 0.008<br><br>Holm-Sidak's multiple comparisons test<br>AcbC vs AcbSh; p = 0.24 (t = 1.201)<br>AcbC vs MPEP; p = 0.07 (t = 2.267)<br>AcbSh vs MPEP; p = 0.007 (t = 3.433)	**One sample t-test - optostim slices**<br>Change from basal (= 1)<br><br><br>AcbC; p = 0.0261 (t = 2.723)<br>AcbSh; p = 0.0054 (t = 3.772)<br>MPEP; p = 0.5030 (t = 0.7061)
**5F** left (Activation masks)	Optostim; n = 9 slices, N = 6 mice	**One-way ANOVA - optostim slices**<br>%Area; F (2, 24) = 9.658, p < 0.001<br><br>Holm-Sidak's multiple comparisons test<br>Glut. Affer. vs Overlap; p = 0.001 (t = 4.024)<br>Glut. Affer. vs Astrocytes; p = 0.63 (t = 0.4822)<br>Overlap vs Astrocytes; p = 0.003 (t = 3.542)	
**5F** right (AcbC-AcbSh bivariate)	Optostim; n = 9 slices, N = 6 mice	**MANOVA - optostim slices**<br>AcbC vs Acbsh<br>d = 0, p = 0.482	
**6B** (Afferents fluorescence)	n = 10 infections, N = 6	**Unpaired t-test Welch-corrected**<br>Two-tailed p value<br>AcbC vs AcbSh; p = 0.01 (t = 3.06)	
**6D** (Affer. Fluo vs EPSCs amplitude)	n = 23 cells, N = 5	**Pearson r correlation**<br>Projection Fluo. Vs EPSCs Amplitude<br>r = 0.5622<br>R squared = 0.316<br>Two-tailed p value; p = 0.005	
**6E** (EPSCs amplitude)	AcbC; n = 4 cells, N = 3 mice<br>AcbSh; n = 21 cells, N = 6 mice	**Unpaired t-test Welch-corrected**<br>Two-tailed p value<br>AcbC vs AcbSh; p < 0.001 (t = 4.362)	
**6H** (Calcium dynamics astrocytes)	AcbC ; n = 28 slices, N = 8 mice<br>AcbSh; n = 28 slices , N = 8 mice<br>MPEP; n = 10 AcbC+AcbSh, 6 slices, N = 2 mice	**One-way ANOVA; Responding ROIs**<br>Responding ROIs; F (2, 63) = 7.783, p < 0.001<br><br>Holm-Sidak's multiple comparisons test<br>AcbC vs AcbSh; p = 0.71 (t = 0.3717)<br>AcbC vs MPEP; p = 0.001 (t = 3.795)<br>AcbSh vs MPEP; p = 0.002 (t = 3.526)<br><br>**One-way ANOVA; Ca^{2+} frequency**<br>Ca^{2+} frequency; F (2, 62) = 6.771, p = 0.002<br><br>Holm-Sidak's multiple comparisons test<br>AcbC vs AcbSh; p = 0.46 (t = 0.7459)<br>AcbC vs MPEP; p = 0.002 (t = 3.637)<br>AcbSh vs MPEP; p = 0.006 (t = 3.117)	**One sample t-test; Responding ROIs**<br>Change from basal (= 1)<br><br><br>AcbC; p < 0.001 (t = 5.45)<br>AcbSh; p < 0.001 (t = 5.22)<br>MPEP; p = 0.0913 (t = 1.89)<br><br>**One sample t-test; Ca^{2+} frequency**<br>Change from basal (= 1)<br><br><br>AcbC; p < 0.001 (t = 6.136)<br>AcbSh; p < 0.001 (t = 4.776)<br>MPEP; p = 0.1413 (t = 1.63)
**7B** (PRQ Glutamatergic afferents)	Optostim; n = 8 slices, N = 6 mice	**Unpaired t-test - optostim slices**<br>Two-tailed p value<br><br>AcbC vs AcbSh; p < 0.001 (t = 8.441)	**One sample t-test - optostim slices**<br>Change from 1<br><br>AcbC; p = 0.0204 (t = 2.896)<br>AcbSh; p < 0.001 (t = 8.706)
**7D** (PRQ Astrocytes)	Basal; n = 8 slices, N = 6 mice<br>Optostim; n = 8 slices, N = 6 mice<br><br>MPEP basal; n = 8 AcbC+AcbSh, 4 slices, N = 2 mice<br>MPEP optostim; n = 8 AcbC+AcbSh, 4 slices, N = 2 mic	**One-way ANOVA - optostim slices**<br>F (2, 21) = 8.532, p = 0.002<br><br>Holm-Sidak's multiple comparisons test<br>AcbC vs AcbSh; p = 0.27 (t = 1.129)<br>AcbC vs MPEP; p = 0.002 (t = 4.006)<br>AcbSh vs MPEP; p = 0.02 (t = 2.876)	**One sample t-test - optostim slices**<br>Change from basal (= 1)<br><br><br>AcbC; p = 0.0026 (t = 4.553)<br>AcbSh; p = 0.0012 (t = 5.215)<br>MPEP; p = 0.9880 (t = 0.01559)
**7F** left (Activation masks)	Optostim; n = 8 slices, N = 6 mice	**One-way ANOVA - optostim slices**<br>%Area;F (2, 21) = 7.075, p = 0.004<br><br>Holm-Sidak's multiple comparisons test<br>Glut. Proj. vs Overlap; p = 0.12 (t = 1.969)<br>Glut. Proj. vs Astrocytes; p = 0.12 (t = 1.792)<br>Overlap vs Astrocytes; p = 0.003 (t = 3.76)	
**7F** right (AcbC-AcbSh bivariate)	Optostim; n = 8 slices, N = 6 mice	**MANOVA - optostim slices**<br>AcbC vs Acbsh<br>d = 1, p = 0.015	

**8B** (Filtered PRQ Astrocytes)	mPFC; n = 9 slices, N = 6 mice<br>Amyg; n = 9 slices, N = 6 mice<br>vHip; n = 8 slices, N = 6 mice	**Two-way ANOVA**<br>mPFC vs Amyg vs vHip; F (2, 46) = 5.089; p = 0.0101<br>AcbC vs AcbSh; F (1, 46) = 0.7246; p = 0.3990<br>**Holm-Sidak's multiple comparisons test**<br>mPFC vs Amyg; p = 0.7358 (t = 0.3394)<br>Amyg vs vHip; p = 0.0153 (t = 2.939)<br>mPFC vs vHip; p = 0.0243 (t = 2.609)	
**8D** (Overlap correlation)	mPFC; n = 9 slices, N = 6 mice<br>Amyg; n = 9 slices, N = 6 mice<br>vHip; n = 8 slices, N = 6 mice	**Pixel-by-pixel Pearson r correlation**<br>mPFC vs Amyg; r = 0.09846; R squared = 0.009694; p = 0.009<br>Amyg vs vHip; r = 0.3158; R squared = 0.09971; p < 0.001<br>mPFC vs vHip; r = -0.2866; R squared =0.08215; p < 0.001	
**8F** (% Overlap)	mPFC; n = 9 slices, N = 6 mice<br>Amyg; n = 9 slices, N = 6 mice<br>vHip; n = 8 slices, N = 6 mice<br>VTA; n = 3 slices, N = 3 slices	**One-way ANOVA**<br>F (3, 25) = 24.49, p < 0.001<br>Holm-Sidak's multiple comparisons test<br>mPFC vs Amyg; p = 0.71 (t = 0.3711)<br>mPFC vs vHip; p = 0.16 (t = 2.012)<br>vHip vs Amyg; p = 0.21 (t = 1.652)<br>mPFC vs VTA; p < 0.001 (t = 8.136)<br>Amyg vs VTA; p < 0.001(t = 7.874)<br>vHip vs VTA; p < 0.001 (t = 6.567)	
**9C** (mPFC + Amyg co-stimulation)	mPFC + Amyg basal (n = 5, N = 3)<br>mPFC + Amyg optostim (n = 5, N = 3)<br><br>mPFC basal; n = 9 slices, N = 6 mice<br>mPFC optostim; n = 9 slices, N = 6 mice<br><br>Amyg basal; n = 9 slices, N = 6 mice<br>Amyg optostim; n = 9 slices, N = 6 mice	**One-way ANOVA; AcbC - optostim**<br>F (2, 20) = 0.9642, p = 0.4<br>Holm-Sidak's multiple comparisons test<br>mPFC + Amyg vs mPFC; p = 0.33 (t = 1.389)<br>mPFC + Amyg vs Amyg; p = 0.39 (t = 0.8836)<br><br>**One-way ANOVA; AcbSh - optostim**<br>F (2, 20) = 3.156, p = 0.06<br>Holm-Sidak's multiple comparisons test<br>mPFC + Amyg vs mPFC; p = 0.05 (t = 2.453)<br>mPFC + Amyg vs Amyg; p = 0.06 (t = 1.993)	**One sample t-test; AcbC - optostim**<br>Change from basal (= 1)<br>mPFC; p = 0.0035 (t = 4.079)<br>mPFC + Amyg; p = 0.4224 (t = 0.8928)<br>Amyg; p = 0.0261 (t = 2.723)<br><br>**One sample t-test; AcbSh - optostim**<br>Change from basal (= 1)<br>mPFC; p = 0.0014 (t = 4.768)<br>mPFC + Amyg; p = 0.1094 (t = 2.053)<br>Amyg; p = 0.0054 (t = 3.772)
**9F** (Amyg + vHip co-stimulation)	Amyg + vHip basal; n = 7 slices, N = 4 mice<br>Amyg + vHip optostim; n = 7 slices, N = 4 mice<br><br>Amyg basal; n = 9 slices, N = 6 mice<br>Amyg optostim; n = 9 slices, N = 6 mice<br><br>vHip basal; n = 8 slices, N = 6 mice<br>vHip optostim; n = 8 slices, N = 6 mice	**One-way ANOVA; AcbC - optostim**<br>F (2, 21) = 4.833, p = 0.02<br>Holm-Sidak's multiple comparisons test<br>Amyg + vHip vs Amyg; p = 0.74 (t = 0.3413)<br>Amyg + vHip vs vHip; p = 0.02 (t = 2.772)<br><br>**One-way ANOVA; AcbSh - optostim**<br>F (2, 21) = 4.601, p = 0.02<br>Holm-Sidak's multiple comparisons test<br>Amyg + vHip vs Amyg; p = 0.04 (t = 2.147)<br>Amyg + vHip vs vHip; p = 0.01 (t = 2.969)	**One sample t-test; AcbC - optostim**<br>Change from basal (= 1)<br>Amyg; p = 0.0261 (t = 2.723)<br>Amyg + vHip; p = 0.3028 (t = 1.127)<br>vHip; p = 0.0026 (t = 4.553)<br><br>**One sample t-test; AcbSh - optostim**<br>Change from basal (= 1)<br>Amyg; p = 0.0054 (t = 3.772)<br>Amyg + vHip; p = 0.5513 (t = 0.631)<br>vHip; p = 0.0012 (t = 5.216)
**9I** (mPFC + vHip co-stimulation)	mPFC + vHip basal; n = 6 slices, N = 5 mice<br>mPFC + vHip optostim; n = 6 slices, N = 5 mice<br><br>mPFC basal; n = 9 slices, N = 6 mice<br>mPFC optostim; n = 9 slices, N = 6 mice<br><br>vHip basal; n = 8 slices, N = 6 mice<br>vHip optostim; n = 8 slices, N = 6 mice	**One-way ANOVA; AcbC - optostim**<br>F (2, 20) = 2.341, p = 0.12<br>Holm-Sidak's multiple comparisons test<br>mPFC + vHip vs mPFC; p = 0.46 (t = 1.145)<br>mPFC + vHip vs vHip; p = 0.46 (t = 0.8184)<br><br>**One-way ANOVA; AcbSh - optostim**<br>F (2, 20) = 0.3209, p = 0.73<br>Holm-Sidak's multiple comparisons test<br>mPFC + vHip vs mPFC; p = 0.68 (t = 0.8011)<br>mPFC + vHip vs vHip; p = 0.68 (t = 0.4656)	**One sample t-test; AcbC - optostim**<br>Change from basal (= 1)<br>mPFC; p = 0.0035 (t = 4.079)<br>mPFC + vHip; p = 0.0393 (t = 2.771)<br>vHip; p = 0.0026 (t = 4.553)<br><br>**One sample t-test; AcbSh - optostim**<br>Change from basal (= 1)<br>mPFC; p = 0.0014 (t = 4.768)<br>mPFC + vHip; p = 0.0463 (t = 2.634)<br>vHip; p = 0.0012 (t = 5.216)
**9L** (mPFC + Amyg + vHip co-stimulation)	mPFC + Amyg + vHip basal; n = 6 slices, N = 3 mice<br>mPFC + Amyg + vHip optostim; n = 6 slices, N = 3 mice<br><br>mPFC basal; n = 9 slices, N = 6 mice<br>mPFC optostim; n = 9 slices, N = 6 mice<br><br>Amyg basal; n = 9 slices, N = 6 mice<br>Amyg optostim; n = 9 slices, N = 6 mice<br><br>vHip basal; n = 8 slices, N = 6 mice<br>vHip optostim; n = 8 slices, N = 6 mice	**One-way ANOVA; AcbC - optostim**<br>F (3, 28) = 6.754, p = 0.001<br>Holm-Sidak's multiple comparisons test<br>mPFC + Amyg + vHip vs mPFC; p = 0.14 (t = 1.858)<br>mPFC + Amyg + vHip vs Amyg; p < 0.001 (t = 4.281)<br>mPFC + Amyg + vHip vs vHip; p = 0.17 (t = 1.405)<br><br>**One-way ANOVA; AcbSh - optostim**<br>F (3, 28) = 8.534, p < 0.001<br>Holm-Sidak's multiple comparisons test<br>mPFC + Amyg + vHip vs mPFC; p < 0.001 (t = 4.287)<br>mPFC + Amyg + vHip vs Amyg; p < 0.001 (t = 4.627)<br>mPFC + Amyg + vHip vs vHip; p < 0.001 (t = 3.801)	**One sample t-test; AcbC - optostim**<br>Change from basal (= 1)<br>mPFC; p = 0.0035 (t = 4.079)<br>Amyg; p = 0.0261 (t = 2.723)<br>vHip; p = 0.0026 (t = 4.553)<br>mPFC + Amyg + vHip; p = 0.8386 (t = 0.2146)<br><br>**One sample t-test; AcbSh - optostim**<br>Change from basal (= 1)<br>mPFC; p = 0.0014 (t = 4.768)<br>Amyg; p = 0.0054 (t = 3.772)<br>vHip; p = 0.0012 (t = 5.216)<br>mPFC + Amyg + vHip; p = 0.0183 (t = 3.445)

SUPPLEMENTARY FIGURES

S2C (Neural excitability)	n = 8 cells, N = 2 mice	**Paired t-test; sEPSCs frequency**<br>Two-tailed p value<br>Before vs After 405 nm; p = 0.53 (t = 0.6536)	**Paired t-test; membrane potential**<br>Two-tailed p value<br>Before vs After 405 nm; p = 0.92 (t = 0.09813)
**S2D** (Calcium dynamics astrocytes)	n = 7 slices, N = 1 mouse	**Paired t-test; Responding ROIs**<br>Two-tailed p value<br>Before vs After 405 nm; p = 0.64 (t = 0.4951)<br><br>**Paired t-test; Ca2+ amplitude**<br>Two-tailed p value<br>Before vs After 405 nm; p = 0.23 (t = 1.322)	**Paired t-test; Ca^{2+} frequency**<br>Two-tailed p value<br>Before vs After 405 nm; p = 0.54 (t = 0. 6447)
**S2F** (Inmunolabeling)	Control Iba1+; n = 31 fields, N = 3 mice<br>405 nm Iba1+; n = 31 fields, N = 3 mice<br><br>Control Iba1+; n = 21 fields, N = 3 mice<br>405 nm Iba1+; n = 24 fields, N = 3 mice<br><br>Control Iba1+; n = 16 fields, N = 2 mice<br>405 nm Iba1+; n = 16 fields, N = 2 mice	**Unpaired t-test; Iba1+**<br>Two-tailed p value<br>Control vs After 405 nm; p = 0.26 (t = 1.138)<br><br>**Unpaired t-test; GFAP+**<br>Two-tailed p value<br>Control vs After 405 nm; p = 0.10 (t = 1.683)	**Unpaired t-test; S100β+**<br>Two-tailed p value<br>Control vs After 405 nm; p = 0.88 (t = 0.1469)
**S3** (Spontaneous photoconversion)	Not infected; n = 9 slices, N = 3 mice<br>$CaMPARI_{GFAP}$; n = 6 slices, N = 3 mice<br>$CaMPARI_{GFAP}$ + violet light; n = 5 slices, N = 3 mice	**One-way ANOVA**<br>F (2, 17) = 24.75, p < 0.001<br><br>Holm-Sidak's multiple comparisons test<br>Not Infected vs $CaMPARI_{GFAP}$; p = 0.06 (t = 1.982)<br>Not Infected vs $CaMPARI_{GFAP}$ + violet light ; p < 0.001 (t = 6.999)<br>$CaMPARI_{GFAP}$ vs $CaMPARI_{GFAP}$ + violet light; p < 0.001 (t = 4.772)	
**S4B** (Intesity curves)	ChR2; n = 9 cells, N = 5 mice<br>ChrimsonR; n = 9 cells, N = 4 mice	**Two-way ANOVA**<br>ChrimsonR vs ChR2; F (1, 79) = 0.022, p = 0.88	
**S4D** (CNQX and D-AP5)	ChR2; n = 8 cells, N = 1 mouse<br>ChrimsonR; n = 10 cells; N = 1 mouse	**Paired t-test; ChrimsonR**<br>Two-tailed p value<br>Basal vs CNQX+D-AP5; p < 0.001 (t = 176.5)	**Paired t-test; ChR2**<br>Two-tailed p value<br>Basal vs CNQX+D-AP5; p < 0.001 (t = 221.3)
**S5A** (Calcium dynamics mPFC)	AcbC; n = 25 slices, N = 8 mice<br>AcbSh; n = 19 slices, N = 8 mice	**Two-way ANOVA; Responding ROIs**<br>AcbC vs AcbSh; F (1, 42) = 1.218, p = 0.28<br>**Paired t-test; Responding ROIs - AcbC**<br>Two-tailed p value<br>Basal vs Optostim; p = 0.01 (t = 2.785)<br><br>**Two-way ANOVA; Ca^{2+} frequency**<br>AcbC vs AcbSh; F (1, 42) = 1.442, p = 0.24<br>**Paired t-test; Ca^{2+} frequency - AcbC**<br>Two-tailed p value<br>Basal vs Optostim; p = 0.02 (t = 2.423)<br><br>**Two-way ANOVA; Ca^{2+} amplitude**<br>AcbC vs AcbSh;F (1, 42) = 1.051, p = 0.31<br>**Paired t-test; Ca^{2+} amplitude - AcbC**<br>Two-tailed p value<br>Basal vs Optostim; p = 0.62 (t = 0.4999)	**Paired t-test; Responding ROIs - AcbSh**<br>Two-tailed p value<br>Basal vs Optostim; p = 0.006 (t = 3.099)<br><br>**Paired t-test; Ca^{2+} frequency - AcbSh**<br>Two-tailed p value<br>Basal vs Optostim; p = 0.02 (t = 2.506)<br><br>**Paired t-test; Ca^{2+} amplitude - AcbSh**<br>Two-tailed p value<br>Basal vs Optostim; p = 0.29 (t = 1.091)
**S5C** (Calcium dynamics Amyg)	AcbC; n = 14 slices, N = 7 mice<br>AcbSh; n = 17 slices, N = 7 mice	**Two-way ANOVA; Responding ROIs**<br>AcbC vs AcbSh; F (1, 29) = 2.318, p = 0.14<br>**Paired t-test; Responding ROIs - AcbC**<br>Two-tailed p value<br>Basal vs Optostim; p = 0.19 (t = 1.394)<br><br>**Two-way ANOVA; Ca^{2+} frequency**<br>AcbC vs AcbSh; F (1, 29) = 1.034, p = 0.32<br>**Paired t-test; Ca^{2+} frequency - AcbC**<br>Two-tailed p value<br>Basal vs Optostim; p = 0.11 (t = 1.712)<br><br>**Two-way ANOVA; Ca^{2+} amplitude**<br>AcbC vs AcbSh; F (1, 29) = 0.2103, p = 0.65<br>**Paired t-test; Ca^{2+} amplitude - AcbC**<br>Two-tailed p value<br>Basal vs Optostim; p = 0.30 (t = 1.079)	**Paired t-test; Responding ROIs - AcbSh**<br>Two-tailed p value<br>Basal vs Optostim; p = 0.01 (t = 2.88)<br><br>**Paired t-test; Ca^{2+} frequency - AcbSh**<br>Two-tailed p value<br>Basal vs Optostim; p = 0.02 (t = 2.48)<br><br>**Paired t-test; Ca^{2+} amplitude - AcbSh**<br>Two-tailed p value<br>Basal vs Optostim; p = 0.89 (t = 0.1385)
**S5E** (Calcium dynamics vHip)	AcbC ; n = 28 slices, N = 8 mice<br>AcbSh; n = 28 slices , N = 8 mice	**Two-way ANOVA; Responding ROIs**<br>AcbC vs AcbSh; F (1, 54) = 4.835, p = 0.03<br>**Paired t-test; Responding ROIs - AcbC**<br>Two-tailed p value<br>Basal vs Optostim; p < 0.001 (t = 7.632)<br><br>**Two-way ANOVA; Ca^{2+} frequency**<br>AcbC vs AcbSh; F (1, 54) = 7.152, p = 0.01<br>**Paired t-test; Ca^{2+} frequency - AcbC**<br>Two-tailed p value<br>Basal vs Optostim; p < 0.001 (t = 6.569)<br><br>**Two-way ANOVA; Ca^{2+} amplitude**<br>AcbC vs AcbSh; F (1, 54) = 0.2787, p = 0.60<br>**Paired t-test; Ca^{2+} amplitude - AcbC**<br>Two-tailed p value<br>Basal vs Optostim; p = 0.22 (t = 1.255)	**Paired t-test; Responding ROIs - AcbSh**<br>Two-tailed p value<br>Basal vs Optostim; p < 0.001 (t = 5.412)<br><br>**Paired t-test; Ca^{2+} frequency - AcbSh**<br>Two-tailed p value<br>Basal vs Optostim; p < 0.001 (t = 4.214)<br><br>**Paired t-test; Ca^{2+} amplitude - AcbSh**<br>Two-tailed p value<br>Basal vs Optostim; p = 0.32 (t = 1.012)

**S7A** (CaMPARI$_{GFAP}$ activation mask)	mPFC basal; n = 9 slices, N = 6 mice<br>mPFC optostim; n = 9 slices, N = 6 mice<br><br><br><br><br><br><br>Amyg basal; n = 9 slices, N = 6 mice<br>Amyg optostim; n = 9 slices, N = 6 mice<br><br><br><br><br><br><br>vHip basal; n = 8 slices, N = 6 mice<br>vHip optostim; n = 8 slices, N = 6 mice	**Two-way ANOVA: mPFC**<br>Basal - Optostim; p < 0.001; F (1, 32) = 51.29<br>Inside - Outside; p < 0.001; F (1, 32) = 14.08<br><br>Holm-Sidak's multiple comparisons test<br>Basal (Inside vs Outside) ; p = 0.28 (t = 1.46)<br>Optostim (Inside vs Outside) ; p = 0.001 (t = 3.847)<br><br>**Two-way ANOVA: Amyg**<br>Basal - Optostim; p < 0.001; F (1, 32) = 23.25<br>Inside - Outside; p = 0.01; F (1, 32) = 7.329<br><br>Holm-Sidak's multiple comparisons test<br>Basal (Inside vs Outside) ; p = 0.69 (t = 0.7827)<br>Optostim (Inside vs Outside) ; p = 0.009 (t = 3.046)<br><br>**Two-way ANOVA: vHip**<br>Basal - Optostim; p < 0.001; F (1, 28) = 37.27<br>Inside - Outside; p < 0.001; F (1, 28) = 16.33<br><br>Holm-Sidak's multiple comparisons test<br>Basal (Inside vs Outside) ; p = 0.36 (t = 1.309)<br>Optostim (Inside vs Outside) ; p < 0.001 (t = 4.405)
**S7B** (Glutamatergic afferents activation mask)	mPFC; n = 9 slices, N = 6 mice<br><br><br><br>Amyg; n = 9 slices, N = 6 mice<br><br><br><br>vHip; n = 8 slices, N = 6 mice	**Paired t-test: mPFC**<br>Two-tailed p value<br>Inside vs Outside; p < 0.001 (t = 5.801)<br><br>**Paired t-test: Amyg**<br>Two-tailed p value<br>Inside vs Outside; p < 0.001 (t = 7.752)<br><br>**Paired t-test: vHip**<br>Two-tailed p value<br>Inside vs Outside; p = 0.003 (t = 4.48)
**S7C** (VTA afferents activation mask)	VTA; n = 3 slices, N = 3 mice	**Paired t-test**<br>Two-tailed p value<br>Inside vs Outside; p = 0.04 (t = 4.959)
**S8D** (TTX PRQ Areas)	Optostim without TTX; n = 9 slices, N = 6 mice<br>Optostim with TTX; n = 4 slices, N = 2 mice	**Two-way ANOVA**<br>Glut. Affer. vs Astrocytes vs Overlap;F (2, 33) = 14.76; p < 0.001<br>without TTX vs with TTX; F (1, 33) = 1.134; p = 0.29<br><br>**Holm-Sidak's multiple comparisons test**<br>Glut. Affer. (without TTX vs with TTX); p = 0.90 (t = 0.6155)<br>Astrocytes (without TTX vs with TTX); p = 0.97 (t = 0.4212)<br>Overlap (without TTX vs with TTX); p = 0.81 (t = 0.808)
**S8E** (TTX PRQ Astrocytes)	Basal without TTX; n = 9 slices, N = 6 mice<br>Optostim without TTX; n = 9 slices, N = 6 mice<br><br>Basal with TTX; n = 4 slices, N = 2 mice<br>Optostim with TTX; n = 4 slices, N = 2 mice	**Two-way ANOVA - optostim**<br>without TTX vs with TTX; F (1, 22) = 0.5983; p = 0.45<br>AcbC vs AcbSh;F (1, 22) = 2.127; p = 0.16
**S9B** (NAc astrocyte density)	n = 15 slices, N = 3 mice	**Unpaired t-test**<br>Two-tailed p value<br>AcbC vs AcbSh; p = 0.61 (t = 0.5125)
**S9E** (NAc astrocyte PRQ distribution)	n = 15 slices, N = 3 mice	**Unpaired t-test**<br>Two-tailed p value<br>AcbC vs AcbSh; p = 0.84 (t = 0.2066)
**S9H** (DHPG stimulation)	basal; n = 7 slices, N = 4 mice<br>DHPG; n = 7 slices, N = 4 mice	**Unpaired t-test - DHPG stim**<br>Two-tailed p value<br>AcbC vs AcbSh; p = 0.64 (t = 0.4852)<br><br>**One sample t-test - DHPG stim**<br>Change from basal (= 1)<br>AcbC; p < 0.001 (t = 6.356)<br>AcbSh; p < 0.001 (t = 8.986)
**S10** (CaMPARI spatial correlations)	mPFC; 736 pixels; n = 9 slices, N = 6 mice<br>Amyg; 736 pixels; n = 9 slices, N = 6 mice<br>vHip; 736 pixels; n = 8 slices, N = 6 mice	**Pixel-by-pixel Pearson r correlation**<br>mPFC vs Amyg; r = 0.5122; R squared = 0.2624; p < 0.001<br>Amyg vs vHip; r = 0.5877; R squared = 0.3454; p < 0.001<br>mPFC vs Amyg; r = 0.705; R squared = 0.4971; p < 0.001
**S11C** (VTA Afferents fluorescence)	n = 6 infections, N = 3 mice	**Unpaired t-test**<br>Two-tailed p value<br>AcbC vs AcbSh; p = 0.38 (t = 0.9137)
**S11E** (VTACalcium dynamics astrocytes)	AcbC; n = 8 slices, N = 5 mice<br>AcbSh; n = 8 slices, N = 5 mice<br>Dop. Antagonist; n = 9 slices, N = 2 mice	**One-way ANOVA; Responding ROIs**<br>Responding ROIs; F (2, 22) = 16.83, p < 0.001<br><br>Holm-Sidak's multiple comparisons test<br>AcbC vs AcbSh; p = 0.20 (t = 1.32)<br>AcbC vs Dop. Ant.; p < 0.001 (t = 5.523)<br>AcbSh vs Dop. Ant.; p < 0.001 (t = 4.165)<br><br>**One sample t-test; Responding ROIs**<br>Change from basal (= 1)<br>AcbC; p = 0.0037 (t = 4.264)<br>AcbSh; p < 0.001 (t = 5.938)<br>Dop. Ant.; p = 0.06 (t = 2.189)<br><br>**One-way ANOVA; Ca^{2+} frequency**<br>Ca^{2+} frequency; F (2, 22) = 12.14, p < 0.001<br><br>Holm-Sidak's multiple comparisons test<br>AcbC vs AcbSh; p = 0.94 (t = 0.082)<br>AcbC vs Dop. Ant.; p < 0.001 (t = 4.266)<br>AcbSh vs Dop. Ant.; p < 0.001 (t = 4.182)<br><br>**One sample t-test; Ca^{2+} frequency**<br>Change from basal (= 1)<br>AcbC; p = 0.0012 (t = 5.236)<br>AcbSh; p = 0.0027 (t = 4.522)<br>Dop. Ant.; p = 0.8883 (t = 0.1449)

**S12B** (VTA PRQ afferents)	Optostim; n = 3 slices, N = 3 mice	**Unpaired t-test - optostim slices**<br>Two-tailed p value<br>AcbC vs AcbSh; p = 0.04 (t = 3.004)	
**S12D** (VTA PRQ Astrocytes)	Basal; n = 3 slices, N = 3 mice<br>Optostim; n = 3 slices, N = 3 mice	**Unpaired t-test - optostim slices**<br>Two-tailed p value<br><br>AcbC vs AcbSh; p = 0.90 (t = 0.138)	**One sample t-test - optostim slices**<br>Change from basal (= 1)<br><br>AcbC; p = 0.0249 (t = 6.217)<br>AcbSh; p = 0.0195 (t = 7.05)
**S12F** left (VTA Activation masks)	Optostim; n = 3 slices, N = 3 mice	**One-way ANOVA - optostim slices**<br>%Area; F (2, 6) = 4.252, p = 0.07<br><br>Holm-Sidak's multiple comparisons test<br>Glut. Proj. vs Overlap; p = 0.16 (t = 2.062)<br>Glut. Proj. vs Astrocytes; p = 0.09 (t = 2.817)<br>Overlap vs Astrocytes; p = 0.48 (t = 0.7556)	
**S12F** right (VTA AcbC-AcbSh bivariate)	Optostim; n = 3 slices, N = 3 mice	**MANOVA - optostim slices**<br>AcbC vs Acbsh<br>d = 0, p = 0.136	
**S13B** (ChrimsonR - tdTom Fluo.)	mPFC; n = 16 infections, N = 8 mice<br>Amyg; n = 13 infections, N = 7 mice<br>vHip; n = 16 infections, N = 8 mice	**One-way ANOVA**<br>F (2, 42) = 0.02261, p = 0.98	
**S13C** (ChR2 - EYFP Fluo.)	mPFC; n = 9 infections, N = 6 mice<br>Amyg; n = 8 infections, N = 6 mice<br>vHip; n = 8 infections, N = 6 mice	**One-way ANOVA**<br>F (2, 22) = 0.002508, p > 0.99	
**S13E** ($CaMPARI_{GFAP}$ Fluo. - Ca^{2+} Imaging)	mPFC; n = 10 infections, N = 5 mice<br>Amyg; n = 10 infections, N = 5 mice<br>vHip; n = 15 infections, N = 8 mice	**Two-way ANOVA**<br>mPFC vs Amyg vs vHip; F (2, 64) = 0.1886, p = 0.83<br>AcbC vs AcbSh; F (1, 64) = 0.506, p = 0.48<br><br>**Holm-Sidak's multiple comparisons test**<br>mPFC (AcbC vs AcbSh); p = 0.85 (t = 0.7333)<br>Amyg (AcbC vs AcbSh); p > 0.99 (t = 0.04819)<br>vHip (AcbC vs AcbSh); p = 0.95 (t = 0.4655)	
**S13F** ($CaMPARI_{GFAP}$ Fluo. - photoconversion)	mPFC; n = 9 infections, N = 6 mice<br>Amyg; n = 8 infections, N = 6 mice<br>vHip; n = 8 infections, N = 6 mice	**Two-way ANOVA**<br>mPFC vs Amyg vs vHip; F (2, 44) = 0.1024, p = 0.90<br>AcbC vs AcbSh; F (1, 44) = 0.07862; p = 0.78<br><br>**Holm-Sidak's multiple comparisons test**<br>mPFC (AcbC vs AcbSh); p > 0.99 (t = 0.02163)<br>Amyg (AcbC vs AcbSh); p > 0.99 (t = 0.2467)<br>vHip (AcbC vs AcbSh); p > 0.99 (t = 0.2502)	
