# Supplementary material for: Ca2+-modulated photoactivatable imaging reveals neuron-astrocyte glutamatergic circuitries within the nucleus accumbens
Source: Nat Commun. 2022 Sep 7;13:5272. doi: 10.1038/s41467-022-33020-6 (PMC9452556; doi:10.1038/s41467-022-33020-6)
Supplement: Supplementary file 3 — Reporting Summary [file 41467_2022_33020_MOESM3_ESM.pdf]

nature portfolio

Corresponding author(s): Marta Navarrete

Last updated by author(s): 2022-14-08

Reporting Summary

Nature Portfolio wishes to improve the reproducibility of the work that we publish. This form provides structure for consistency and transparency in reporting. For further information on Nature Portfolio policies, see our Editorial Policies and the Editorial Policy Checklist.

Statistics

For all statistical analyses, confirm that the following items are present in the figure legend, table legend, main text, or Methods section.

n/a	Confirmed	
☐	☒	The exact sample size (*n*) for each experimental group/condition, given as a discrete number and unit of measurement
☐	☒	A statement on whether measurements were taken from distinct samples or whether the same sample was measured repeatedly
☐	☒	The statistical test(s) used AND whether they are one- or two-sided<br>*Only common tests should be described solely by name; describe more complex techniques in the Methods section.*
☐	☒	A description of all covariates tested
☐	☒	A description of any assumptions or corrections, such as tests of normality and adjustment for multiple comparisons
☐	☒	A full description of the statistical parameters including central tendency (e.g. means) or other basic estimates (e.g. regression coefficient) AND variation (e.g. standard deviation) or associated estimates of uncertainty (e.g. confidence intervals)
☐	☒	For null hypothesis testing, the test statistic (e.g. *F*, *t*, *r*) with confidence intervals, effect sizes, degrees of freedom and *P* value noted<br>*Give P values as exact values whenever suitable.*
☒	☐	For Bayesian analysis, information on the choice of priors and Markov chain Monte Carlo settings
☒	☐	For hierarchical and complex designs, identification of the appropriate level for tests and full reporting of outcomes
☒	☐	Estimates of effect sizes (e.g. Cohen's *d*, Pearson's *r*), indicating how they were calculated

Our web collection on statistics for biologists contains articles on many of the points above.

Software and code

Policy information about availability of computer code

Data collection	Electrophysiological data was collected using PC_ONE amplifiers (Dagan) and pClamp software v.10.7 (Molecular Devices).<br>Imaging data was collected with a Leica SP-5 inverted confocal microscope using Leica LAS AF software v2.7.3.9723.
Data analysis	MATLAB software (R2018a; Mathworks, Natick, MA, USA) was used for calcium imaging, Partition in Regular Quadrants (PRQ) spatial anaysis and statical diffference calculations.<br>Image J Software (public domain software developed at the US NIH) was used for afferent density and opsin transfection analysis, calcium imaging analysis and PRQ analysis.<br>Cell Profiler software v.4.0.6. (public domain software developed at the Broad Institute) was used for automatized cell quantification.<br>GraphPad Prism software v.7.0 was used for statistical analysis.<br>Custom code for PRQ analysis is available at https://github.com/JulioEI/PRQ

For manuscripts utilizing custom algorithms or software that are central to the research but not yet described in published literature, software must be made available to editors and reviewers. We strongly encourage code deposition in a community repository (e.g. GitHub). See the Nature Portfolio guidelines for submitting code & software for further information.

Data

Policy information about availability of data

All manuscripts must include a data availability statement. This statement should provide the following information, where applicable:

- Accession codes, unique identifiers, or web links for publicly available datasets
- A description of any restrictions on data availability
- For clinical datasets or third party data, please ensure that the statement adheres to our policy

All data generated in this study are provided in the Supplementary Information/Source Data file. Code is available at https://github.com/JulioEI/PRQ

Field-specific reporting

Please select the one below that is the best fit for your research. If you are not sure, read the appropriate sections before making your selection.

- [x] Life sciences
- [ ] Behavioural & social sciences
- [ ] Ecological, evolutionary & environmental sciences

For a reference copy of the document with all sections, see nature.com/documents/nr-reporting-summary-flat.pdf

Life sciences study design

All studies must disclose on these points even when the disclosure is negative.

Sample size	We did not perform power analysis to predetermine sample sizes, but our sample size were determined based on obtaining large enough data points per condition to test for normality of distribution and perform appropriate statistical analysis.
Data exclusions	For all experiments, the animals' brain were processed for histology to confirm virus infection sites. Data will be excluded if animals display an inaccurate viral injection site.<br>Electrophysiology recordings were considered when the series and input resistances, resting membrane potential and stimulus artefact duration did not change > 20%.
Replication	All experiments were conducted at least three times independently, obtaining results that were consistent to guarantee reproducibility. Detailed methods and description of protocols of the experiments will allow for<br>coherent reproduction.
Randomization	For all experiments, male and female mice were randomly used. For monitoring in real-time Ca2+ signals same slices were used for basal and optostimulated conditions. For photoconversion experiments, NAc consecutive slices at coordinates AP +1.3 mm and AP + 0.98 mm were used to determine basal and optostimulated experimental groups, being paired for posterior analysis. These AP coordinates were alterned between basal-optostimulation in different hemispheres.
Blinding	Collection and analysis of electrophysiological data were performed blind to the condition of the experiments. Data analysis were performed by different investigators and analysis to avoid conscious and unconscious bias.

Reporting for specific materials, systems and methods

We require information from authors about some types of materials, experimental systems and methods used in many studies. Here, indicate whether each material, system or method listed is relevant to your study. If you are not sure if a list item applies to your research, read the appropriate section before selecting a response.

Materials & experimental systems

n/a	Involved in the study	
☐	☒	Antibodies
☒	☐	Eukaryotic cell lines
☒	☐	Palaeontology and archaeology
☐	☒	Animals and other organisms
☒	☐	Human research participants
☒	☐	Clinical data
☒	☐	Dual use research of concern

Methods

n/a	Involved in the study	
☒	☐	ChIP-seq
☒	☐	Flow cytometry
☒	☐	MRI-based neuroimaging

Antibodies

Antibodies used	anti-S100 (ab868, Abcam)<br>anti-NeuN (MAB377, Merck)

anti-S100β (287003, Synaptic Systems)
anti-GFAP (G3893, Sigma)
anti-Iba1 (234004, Synaptic Systems)
anti-mouse Alexa 647 (A21236, Thermo Fisher Scientific)
anti-rabbit Alexa 405 (A31556, Thermo Fisher Scientific)
anti-mouse Alexa 488 (A11001, Invitrogen)
anti-guinea pig Alexa 647 (A21450, Invitrogen)

Validation	All antibodies are validated for species by manufacturer. Anti-S100 (ab868) has been validated to recognise the protein S100 with immunohistochemistry in the literature (PMID: 25184527). Anti-NeuN (MAB3377) has been validated for immunohistochemistry using the NeuN knock-out mice (PMID: 29398366). Anti-S100B (287003) has been validated and used to detect S100B with immunohistochemistry (PMID: 33674568; PMID: 34010654). Anti-GFAP (G3893) monoclonal antibody reacts specifically with GFAP in immunoblotting assays and labels astrocytes in immunohistochemical staining and was previous validated by us (PMID: 31273206) and the literature (PMID: 31645758). Anti-Iba1 (234004) polyclonal antibody has been previous validated in the literature (PMID:26863192; PMID:30177815).

Animals and other organisms

Policy information about studies involving animals; ARRIVE guidelines recommended for reporting animal research

Laboratory animals	C57BL/6J wild-type mice of both sexes were used and were housed in standard laboratory cages with ad libitum access to food and water, under a 12:12 h dark-light cycle in temperature and humidity-controlled rooms (21-24 °C and 45-65% respectively). All animals were 1-3 months old during the experiments.
Wild animals	The study did not involve wild animals.
Field-collected samples	No field-collected samples were used.
Ethics oversight	All experimental procedures with animals will be carried out in accordance with the protocols approved by the Cajal Institute and the Community of Madrid (PROEX 40/18). The protocols that we use try to minimize pain and suffering of the animals. The Animal Service of the Cajal Institute (registration number ES280790000184) complies with all the recommendations on accommodation of the Council of Europe and the current regulations for Testing and Protection of Animals used for Experimental and other Scientific Purposes reflected in Directive 2010/63/EU.

Note that full information on the approval of the study protocol must also be provided in the manuscript.
